# Sex differences in cognitive flexibility are driven by the estrous cycle and stress-dependent

**DOI:** 10.3389/fnbeh.2022.958301

**Published:** 2022-08-04

**Authors:** Andrew T. Gargiulo, Jiayin Hu, Isabella C. Ravaglia, Annie Hawks, Xinyue Li, Katherine Sweasy, Laura Grafe

**Affiliations:** Grafe Lab, Department of Psychology, Bryn Mawr College, Bryn Mawr, PA, United States

**Keywords:** stress, cognitive flexibility, sex differences, orexin, estrogen

## Abstract

Stress is associated with psychiatric disorders such as post-traumatic stress disorder, major depressive disorder, anxiety disorders, and panic disorders. Women are more likely to be diagnosed with these stress-related psychiatric disorders than men. A key phenotype in stress-related psychiatric disorders is impairment in cognitive flexibility, which is the ability to develop new strategies to respond to different patterns in the environment. Because gonadal hormones can contribute to sex differences in response to stress, it is important to consider where females are in their cycle when exposed to stress and cognitive flexibility testing. Moreover, identifying neural correlates involved in cognitive flexibility could not only build our understanding of the biological mechanisms behind this crucial skill but also leads to more targeted treatments for psychiatric disorders. Although previous studies have separately examined sex differences in cognitive flexibility, stress effects on cognitive flexibility, and the effect of gonadal hormones on cognitive flexibility, many of the findings were inconsistent, and the role of the estrous cycle in stress-induced impacts on cognitive flexibility is still unknown. This study explored potential sex differences in cognitive flexibility using an operant strategy shifting-paradigm after either control conditions or restraint stress in freely cycling female and male rats (with estrous cycle tracking in the female rats). In addition, we examined potential neural correlates for any sex differences observed. In short, we found that stress impaired certain aspects of cognitive flexibility and that there were sex differences in cognitive flexibility that were driven by the estrous cycle. Specifically, stress increased latency to first press and trials to criterion in particular tasks. The female rats demonstrated more omissions and perseverative errors than the male rats; the sex differences were mostly driven by proestrus female rats. Interestingly, the number of orexinergic neurons was higher in proestrus female rats than in the male rats under control conditions. Moreover, orexin neural count was positively correlated with number of perseverative errors made in cognitive flexibility testing. In sum, there are sex differences in cognitive flexibility that are driven by the estrous cycle and are stress-dependent, and orexin neurons may underlie some of the sex differences observed.

## Introduction

Stress is associated with a variety of psychiatric disorders such as post-traumatic stress disorder (PTSD), major depressive disorder (MDD), anxiety disorders, and panic disorders (Carr et al., 2013; Bangasser and Valentino, 2014). Interestingly, women are more likely to be diagnosed and often have different symptoms and severity of stress-related psychiatric disorders than men (Nestler et al., 2002; Keane et al., 2006; Bangasser and Valentino, 2014; Swaab and Bao, 2020). Therefore, investigating sex differences and underlying biological mechanisms is crucial to the diagnosis and treatment of these disorders.

A key phenotype in stress-related psychiatric disorders is cognitive impairment (Ben-Zion et al., 2018; Boisseau and Garnaat, 2018; Doss et al., 2021). Importantly, stress is related to cognitive deficits such as impaired cognitive flexibility (Powell and Ragozzino, 2017). Cognitive flexibility is the ability to change or switch between mental sets and develop new strategies to adapt and respond to different patterns in the environment (Powell and Ragozzino, 2017; Irwin et al., 2019). Thus, this is an important skill for daily functioning. Understanding more about how stress affects the brain and cognition is vital in treating stress-related psychiatric disorders.

Although our ultimate goal is to understand how stress affects humans, studying stress in animal models allows for control of more variables. Importantly, there are shared physiological and behavioral responses to stress in both humans and rodents, which makes studying the stress response in rodents translatable to humans (Schöner et al., 2017). Among all stress protocols in rodents, restraint stress is one of the simplest and most common approaches (Campos et al., 2013; Taslimi et al., 2019). This study examines how acute restraint stress affects cognitive flexibility performance in male and female Sprague Dawley rats in order to better understand the biological underpinnings of any sex differences observed.

Previous research has assessed cognitive flexibility using attentional set-shifting paradigms in both humans and animals (Brown and Tait, 2016). The paradigms are focused on flexibility in shifting between thoughts and actions and involve problem-solving and exploration (Ionescu, 2012; Marko and Riečanský, 2018). In humans, the Wisconsin Card Sorting Test (WCST) is a common test for cognitive flexibility, while in rodents, the Attentional Set Shifting Paradigm is the most common (Brown and Tait, 2016; Miles et al., 2021). In both procedures, intradimensional (ID) and extradimensional (ED) cues are used, and the subject must learn to shift both within and between dimensions to complete the test (Brown and Tait, 2016).

In this study, an automated operant strategy-shifting paradigm is used in which the dimensions are lever positions and light cues; rodents must undergo initial discrimination between the levers, followed by reversal of the levers and, lastly, an extradimensional shift to light cues (Floresco et al., 2008; Hurtubise and Howland, 2016; Grafe et al., 2017a; Gargiulo et al., 2020). Reversal learning involves a change in response strategy in the same stimulus dimension (e.g., lever position), actively suppressing a previously learned response strategy while acquiring a new competing strategy. The extradimensional shift (sometimes referred to as “strategy shifting” or “set shifting”) also requires changing a response strategy and suppressing a previously learned rule, but it is across stimulus dimensions (e.g., from lever position to a light cue) (Brady and Floresco, 2015). Both of these tasks require high-level cognitive processes that are necessary for behavioral flexibility and are mediated by unique subregions in the frontal cortex (Brown and Tait, 2016). Most previous research studies on adults indicate that the extradimensional shift is more difficult than reversal learning (Buss, 1956; Harrow and Friedman, 1958). Importantly, this operant strategy-shifting paradigm also allows for assessment of different kinds of errors, including perseverative errors, which are persistent responses made by a subject on the basis of a previous rule, and these error types are common in stress-related psychiatric disorders, demonstrating cognitive rigidity and an inability to adapt to change (Uddo et al., 1993; Vasterling et al., 1998; Van Laethem et al., 2016; Miles et al., 2021). Moreover, this behavioral paradigm allows for quantification of omissions, which are failures to respond to a cue and appear to be more common in patients with PTSD, indicating slower cortical processing and attentional deficits (Korgaonkar et al., 2021). Thus, this paradigm is translationally relevant.

Thus far, there have been limited studies exploring sex differences in cognitive flexibility, with the majority of research indicating that women require more trials for reversal learning compared to men, but that there were no sex differences in ED shifts (LaClair et al., 2019; Hilz et al., 2022). Moreover, studies exploring the effects of stress on cognitive flexibility tend to only include male subjects and are equivocal in their findings. In short, studies have demonstrated that the effects of stress on cognitive flexibility vary depending on the length of the stress (with chronic stress showing greater impairments in cognitive flexibility than acute stress), stages of the task (some stress affects ID tasks while others affect ED tasks), and the time after stress (with both long-term or short-term effects) (Thai et al., 2013; Hurtubise and Howland, 2016; Sullivan et al., 2019). Sex differences have been observed in the effects of stress on cognitive flexibility in some studies, although the findings have also been inconsistent between animal and human research. For example, rodent research indicates that stress impairs cognitive flexibility more strongly in females, and human research indicates that men are more susceptible to cognitive flexibility deficits after stress (Shields et al., 2016; Goldfarb et al., 2017; Grafe et al., 2017a). Research studies measuring other aspects of cognition (including spatial memory) indicate that chronic restraint stress may enhance performance in women compared with men, although sex differences may be task-dependent and are heavily influenced by the phase of the light cycle (Bowman et al., 2009; Huynh et al., 2011; Peay et al., 2020). These changes in cognition after restraint stress stem from sex-specific changes in the brain (Conrad et al., 2017). The inconsistency between findings from different studies indicate that more research is needed to investigate sex differences in the effects of stress on cognitive flexibility and to explore the underlying mechanisms for a more comprehensive understanding.

Although previous research has explored the effect of repeated restraint stress on cognitive flexibility, the experimental paradigm included the effect of restraint stress on learning (e.g., 5 consecutive days of repeated restraint was administered, followed by 3 days of training for the cognitive flexibility task; finally, the cognitive flexibility test was administered) (Grafe et al., 2017a). Conversely, in this study, the training for cognitive flexibility was conducted for 3 days, and on the 4th day, acute restraint was administered immediately prior to the cognitive flexibility testing. Thus, in this study, we are examining how stress affects cognitive flexibility performance and eliminating the effect that stress may have on learning the task.

Because gonadal hormones can contribute to sex differences in response to stress (Becker et al., 2005; Oyola and Handa, 2017; Heck and Handa, 2019), it is important to consider where women are in their cycle when exposed to stress and cognitive flexibility testing. In general, estrogen has been shown to promote stress response, while progesterone inhibits the stress response (Becker et al., 2005; Oyola and Handa, 2017; Heck and Handa, 2019). In rodents, the reproductive cycle is called the estrous cycle, which happens for 4 to 5 days (Becker et al., 2005) and includes the diestrus, proestrus, and estrus phases (Becker et al., 2005; Oyola and Handa, 2017). The diestrus in rodents is equivalent to the follicular phase in humans when estrogen gradually increases. In the proestrus phase, both estrogen and progesterone levels increase (progesterone starts to increase later than estrogen) and peak before ovulation. The estrous phase is when ovulation occurs and estrogen and progesterone begin to decline (Becker et al., 2005; Oyola and Handa, 2017). Previous studies have separated these phases into low and high gonadal hormone phases (i.e., diestrus/estrus vs. proestrus) (Goldman et al., 2009). Importantly, when estrogen is lowest during the diestrus phase, female rodents are observed to secrete stress hormones in a similar manner to male rodents (Heck and Handa, 2019). In contrast, stress hormones are higher in proestrus than in diestrus (Oyola and Handa, 2017; Heck and Handa, 2019).

There have been inconsistent findings on the impacts of gonadal hormones on cognitive flexibility. For example, one rodent study has found that 17β-estradiol treatment in females leads to worse set-shifting performance compared to both males and ovariectomized control females (Hilz et al., 2022). However, another rodent study has shown that in ovariectomized female rats, 17β-estradiol treatment results in poorer learning in simple discrimination but improved learning in extradimensional set-shifting (Lipatova et al., 2016). Furthermore, in male rats, high testosterone has been reported to impair both extradimensional set-shifting and reversal learning (Wallin and Wood, 2015; Tomm et al., 2022). Thus, more research is necessary to better understand how changes in gonadal hormones may impact cognitive flexibility. Additionally, studies that consider how stress affects cognitive flexibility while considering gonadal hormone status are nonexistent, which is the reason for this study.

Identifying neural correlates involved in cognitive flexibility could not only build our understanding of biological mechanisms behind this crucial skill but also leads to more targeted treatments for psychiatric disorders associated with impairments of cognitive flexibility. Many brain regions are involved in cognitive flexibility. Here, we focus on two regions: the prefrontal cortex and the lateral hypothalamus, which have been shown to play a role in stress-induced changes in cognitive flexibility (McAlonan and Brown, 2003; Placek et al., 2013; Arnsten, 2015; Lipatova et al., 2016; Grafe et al., 2017a; Durairaja and Fendt, 2021). Importantly, previous research has indicated that the orbital prefrontal cortex (OFC) is important for reversal learning (McAlonan and Brown, 2003), whereas the medial prefrontal cortex (mPFC) is required for extradimensional set-shifting (Placek et al., 2013; Lipatova et al., 2016; Durairaja and Fendt, 2021). Moreover, orexin neuropeptides produced in the lateral hypothalamus have been shown to impair reversal learning both in control and stressed animals (Grafe et al., 2017a; Durairaja and Fendt, 2021). Hormonal fluctuations associated with the estrous cycle can alter both prefrontal and orexin activities (Porkka-Heiskanen et al., 2004; Duclot and Kabbaj, 2015). Thus, understanding the effects of stress on these brain regions and sex differences in the effects (especially sex differences mediated by the estrous cycle) may help us develop sex-specific treatments for stress-related disorders.

This study explored potential sex differences in cognitive flexibility using an operant strategy-shifting paradigm (Floresco et al., 2008; Grafe et al., 2017a; Gargiulo et al., 2020) after either control conditions or acute restraint stress in freely cycling female and male rats (with estrous cycle tracking in the female rats). In addition, we examined both the PFC and orexin neurons in the lateral hypothalamus as potential neural correlates for any sex differences observed. In short, we found that there are sex differences in cognitive flexibility that are driven by the estrous cycle and are stress-dependent, and that orexin neurons in the lateral hypothalamus may underlie some of the sex differences observed. As cognitive flexibility is affected in many stress-related psychiatric disorders, better understanding how stress and the estrous cycle affect this phenotype at the neurobiological level is important in individualized diagnosis and treatment of these disorders.

## Methods

### Subjects and overview of procedures

**Figure 1:**
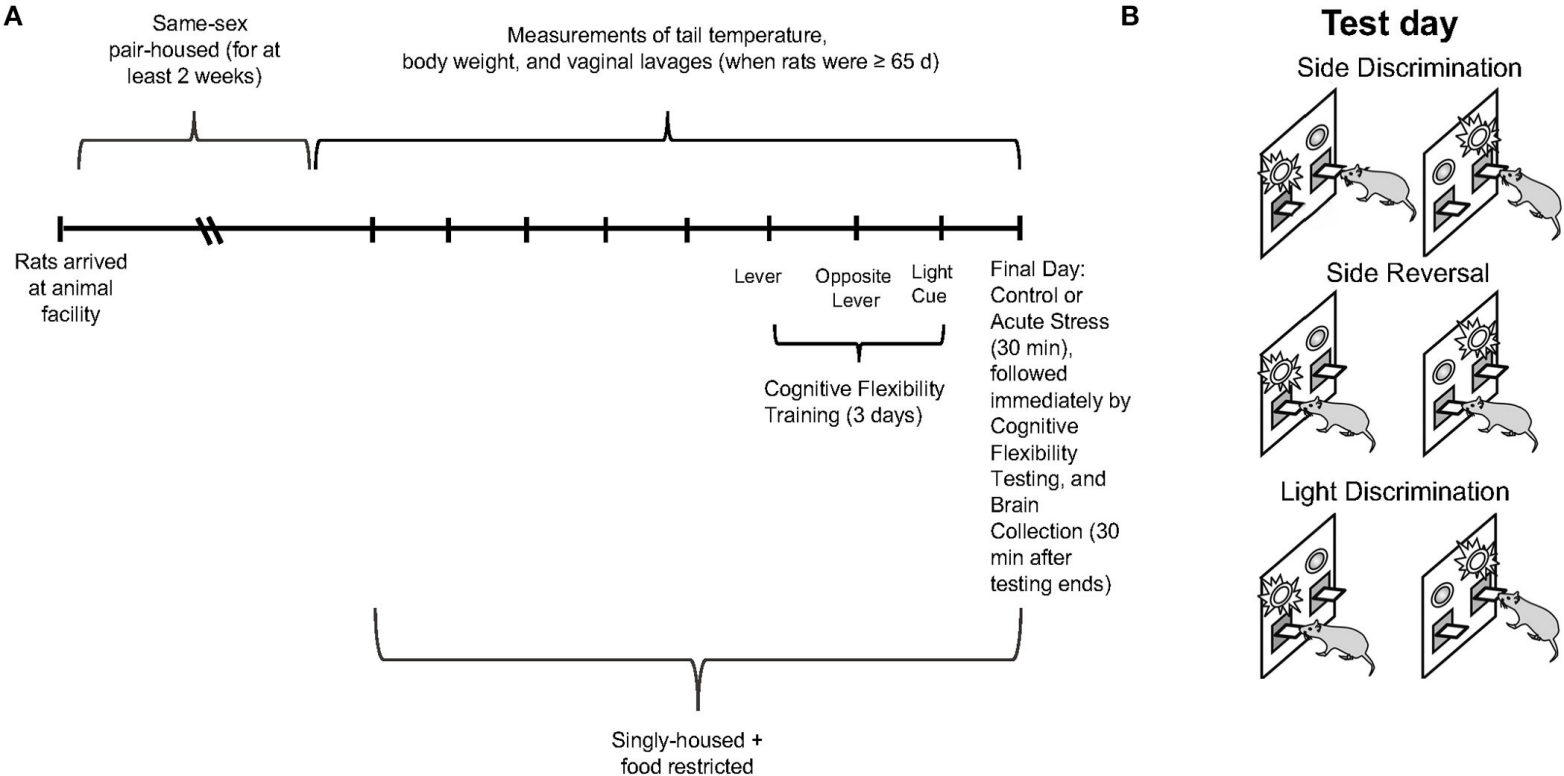
**(A)** Experimental paradigm depicting the timeline for collection of tail temperature/body weight/vaginal lavage measures, operant strategy shifting training/testing, restraint stress, and brain collection. **(B)** Schematic illustrating the operant set shifting paradigm on test day.

Sprague-Dawley rats (*n* = 24 male and *n* = 40 female; Envigo, Indianapolis, IN; run in 4 cohorts) were same-sex pair-housed and accommodated in a 12-h light/dark cycle with lights on and off at 7am and 7pm, respectively. The rats were acclimated to the animal facility for at least 2 weeks after arrival. Food and water were available *ad libitum*. When the rats were at least 65 days of age, daily measurements of tail temperature (using infrared technology, IR Rodent Thermometer; BIOSEB, Vitrolles, France), body weight, and vaginal lavage (in the female rats only) were conducted until the conclusion of the experiment (see Figure 1A for experimental timeline). Thus, the male and female rats were handled for a comparable amount of time, which has been reported to affect behavioral performance (Bohacek and Daniel, 2007). However, we did not perform anal swabs on males as a control for the vaginal swab procedure in females, as some previous studies have (Talboom et al., 2014). Tail temperature was not significantly different between the male and female rats over the course of the study; therefore, data are not shown. Five days before operant strategy-shifting training, the animals were singly housed and underwent food restriction. During food restriction, the rats were restricted to 80% of free-feeding weight as a guideline; water was still available *ad libitum* (Gargiulo et al., 2020). Daily body weights were collected to ensure that the rats were gaining weight according to Sprague Dawley growth data (Envigo, Indianapolis, IN) and did not lose any weight during food restriction for the operant strategy shifting paradigm; the data are not shown. The entire operant strategy-shifting paradigm lasted for 4 days, including 3 days of training and 1 day of testing. On test day, the rats were randomly divided into two conditions: control (*n* = 12 males and *n* = 20 females) and 30-min restraint stress (*n* = 12 males and *n* = 20 females). Restraint video recordings for 2 of the stressed females were not fully captured (beginning minute of restraint was missing), so they could not be analyzed. Thus, *n* = 18 for the female restraint stress data rather than *n* = 20. Thirty minutes after the operant strategy-shifting task ended, the rats were sacrificed, and their brains were collected for slicing and staining procedures. Three male rats did not learn the operant strategy-shifting task, thus, total *n* = 21 for male behavioral data rather than *n* = 24. The first cohort of rats could not be analyzed for c-fos as the PFC slices were not in good-enough condition for quantification; thus, the n for c-fos data is 16 for the male and 32 for the female rats. All the procedures were approved by the Institutional Animal Care and Use Committee (IACUC) at Bryn Mawr College.

### Procedures

#### Lavage

When the female rates were at least 65 days of age, vaginal lavage was performed every morning to track the estrous cycle until the end of the experiment, as previously described (Gargiulo et al., 2020). Briefly, a glass pipette with warm water inside was pressed and released several times on the surface of the vaginal orifices of the female rats. The sample was then transferred to a specimen slide with acrylic paint circles and was observed under a Leica microscope (DM4 B; Leica Camera, Wetzlar, Germany) at 5× magnification. The estrous cycle phases from the vaginal cytology samples were determined as previously described (Becker et al., 2005). In short, samples that displayed predominantly leukocytes (and some larger round cells without nuclei) were categorized as diestrus. Samples that had primarily nucleated epithelial cells were categorized as proestrus. Samples that principally included cornified cells were categorized as estrus. Representative images of vaginal lavage samples categorized into each phase of the estrous cycle are included in Supplementary Figure 1.

#### Restraint stress

The 12 male and 20 female rats randomly assigned to the stress condition were exposed to a single acute restraint stress just prior to the operant strategy-shifting test. Briefly, the rats were placed in a Broome-style transparent restraint tube for 30 min. Previous research has indicated that corticosterone in the plasma increases significantly by 15 min of restraint and peaks at 30 min of restraint; thus, this duration of restraint is sufficient to induce a reliable stress response (Jaferi et al., 2003). A camera recorded the time it took the experimenter to restrain a rat as well as the struggle behavior displayed by the rodent for the first 10 min of restraint. Time to restrain was defined as the time it took the experimenter to secure the rodent in the restrainer (in seconds). Struggle behavior was quantified as cumulative duration of movement (in seconds); this was hand-coded by an experimenter blind to other experimental conditions (e.g., sex or estrous phase). Total stress behavior was quantified as the summation of time to restrain and struggle behavior. The detailed steps of the restraint stress procedure are as previously described (Grafe et al., 2017a; Gargiulo et al., 2020). After the stress exposure, the rats were immediately transferred into the operant chamber to perform the operant strategy-shifting test.

#### Operant strategy-shifting paradigm

The operant strategy-shifting paradigm spans 4 days. For both training and tests, each rat was placed in the operant chamber with a house light, two retractable levers with two stimulus lights above them, and a food pellet dispenser for reinforcement for these tasks (MED-PC, St. Albans, VT, United States). The training was completed during the first 3 days (each rat first learned to press the right lever on day 1, left lever on day 2, and respond to light cues above the levers on day 3). For each day of training, 50 correct trials had to be completed before the training ended. There were no light cues on the first 2 days of the training. For day 3 of the training, stimulus lights appeared above both levers for 5 s, signaling that the rat should make a response; the correct lever (with food pellet reward) was chosen randomly for each trial. If the rat did not press a lever when the lights were on, the house light and stimulus lights were turned off for 5 s, and no food pellet was delivered; this lack of response is defined as an omission. If the rat pressed a lever when the stimulus lights were on and the side was randomly assigned to be the correct side, the house light and stimulus lights stayed on for 3 s, while a food pellet was delivered, and then all the lights were turned off for 7 s before the next trial started; this is defined as a correct response. If the rat pressed a lever when the lights were on and the side was randomly assigned to be the incorrect side, the house light and stimulus lights were turned off for 10 s, and no food pellet was delivered; this is defined as an error. These conditions were matched during testing. Side bias for a particular lever was calculated after day 3 of training, and the rats were started on their least preferred side on test day.

On the 4th day (test day), the rats underwent the three tasks consecutively, where a light cue would appear above one lever for each trial (see Figure 1B for schematic). Briefly, the test day began with side discrimination, where the rats learned to press the lever on one side of the operant chamber to be rewarded regardless of where the light cue was illuminated, followed by side reversal, also called reversal learning, where the rats learned to press the lever on the other side of the operant chamber to be rewarded regardless of where the light cue was illuminated and, finally, light discrimination, also called extradimensional set-shifting or set-shifting, where the rats learned to press the lever under the illuminated light cue to be rewarded (Floresco et al., 2008; Hurtubise and Howland, 2016; Grafe et al., 2017a; Gargiulo et al., 2020). Each rat had to press the correct lever 8 times consecutively to complete each task. The data for the number of correct responses, errors (including types of errors), omissions, and latency to first press were collected with the computer software that operated the chamber. Error types were characterized by logistic regression to determine if the rats perseverated on a previous rule (perseverative error) or failed to acquire or maintain a new rule (regressive error) as described previously (Snyder et al., 2014). Briefly, every trial attempted was categorized as correct or incorrect and regressed by trial number. A logistic curve of best fit was generated, and the trial number after which the value of the curve became greater than or equal to chance performance value of 50% was noted. Errors that occurred before this trial were categorized as perseverative, and errors that occurred after were categorized as regressive. More details of the procedures for the training and testing phases have been previously described (Gargiulo et al., 2020).

#### Immunohistochemistry

All the rats were sacrificed by rapid decapitation 30 min after they finished the light discrimination task (the final task for test day). Their brains were extracted and submerged in 4% paraformaldehyde for 3 days and then transferred to and stored in 30% sucrose. Each brain was sectioned into 40-μm slices using a freezing Leica microtome (SM2000R; Leica Microsystems, Wetzlar, Germany) and stored on 12-well plates with cryoprotectant in each well. The first series of tissues (Bregma 3.7 to −0.26 mm) was stained for *c-fos* expression in the prefrontal cortex, and the third series of tissues (Bregma, 2.3to −4.3 mm) was stained for cells that express orexin in the lateral hypothalamus.

##### *C-fos* in the PFC

The detailed immunochemical staining procedures for *c-fos* in the PFC and OFC took 2 days as previously described (Gargiulo et al., 2020). In short, we incubated slices in a mouse anti-c-fos primary antibody solution (1:500; ab208942, Abcam, Cambridge, United Kingdom) with 3% normal donkey serum (NDS) and phosphate-buffered saline with Triton-X (PBS/Tx) overnight at room temperature. On the 2nd day, we incubated the slices in the biotin-SP-conjugated donkey anti-mouse secondary antibody (1:250; 715-065-150; Jackson ImmunoResearch, West Grove, United States) with 3% NDS and PBS/Tx for 2 h at room temperature, followed by the avidin biotin peroxidase complex, and 3,3'-diaminobenzidine (DAB) solution. *C-fos* expression was quantified in the prelimbic cortex (PRL), infralimbic cortex (IL), and orbitofrontal cortex (OFC). The bregma levels of the images were from 2.2 to 3.7 mm for the IL and 3.2 to 4.7 mm for the PRL and the OFC. For each rat, an average of 8 pictures per brain area was analyzed. The *c-fos* expression for each image was counted with a macro using ImageJ, with a rolling radius of 40, a Gaussian Blur sigma of 5 and 0.75, a particle size of.02-10, and a particle circularity of.5-1. The *c-fos* counts for each bregma level were determined by averaging the counts across all the images (both left and right hemispheres) within that bregma level. The final *c-fos* expression for each PFC subregion in each rat was determined by averaging counts across all the bregma levels analyzed for that region.

##### Orexin in the lateral hypothalamus

The immunofluorescence procedure for staining orexin in the lateral hypothalamus took 2 days as previously described (Grafe et al., 2017a; Gargiulo et al., 2021). Briefly, on the 1st day, slices were incubated in a rabbit anti-orexin-A primary antibody solution (1:500, ab255294; Abcam, Cambridge, United Kingdom) with 3% NDS and PBS/Tx overnight at room temperature. On the 2nd day, we incubated the tissue in donkey an anti-rabbit Alexa Fluor 488 secondary antibody (1:500, a-21206; Thermo Fisher Scientific, Waltham, United States) with 3% NDS and PBS/Tx for 1 h at room temperature. Orexin-expressing cells in the lateral hypothalamus were hand-counted using the Multi-point Tool in ImageJ. The bregma levels of the images were from −1.3 to −4.52mm. An average of 22 images was analyzed for each rat. The orexin cell counts within each bregma level were determined by averaging the counts across both the left and right hemispheres. The final number of cells expressing orexin was determined by averaging counts across all the bregma levels analyzed in each rat.

### Statistical analysis

All the data are presented as mean ± standard error of the mean. The Graphpad prism software (version 9.3.1) was employed for statistical analysis. Outliers were identified as 2 standard deviations above or below the mean. Independent sample *t*-tests (Student's *t*-test for groups with homogeneity of variances and Welch's *t*-test for groups without homogeneity of variances) were conducted to examine the relationship between sex and task performance for each task during the training phase. Task performance during training was assessed with Trials to criterion, Errors, Time to criterion, and Latency to first press for each task (plus Omission for learning to respond to light cue). A one-way ANOVA followed by the Tukey's *post-hoc* tests was conducted to analyze the relationship between sex (male vs. female rats) and stress (control vs. stress) on behaviors during restraint (measured by Time to Restrain, Struggle Behavior, and Total Stress Behavior). Two-way ANOVAs followed by Tukey's *post-hoc* tests were conducted to examine the relationship between sex (male vs. female rats) and stress (control vs. stress) for each task during the testing phase. To analyze gonadal hormone status, we separated the female rats into low vs. high gonadal hormone groups (i.e., diestrus/estrus vs. proestrus), as previously described (Goldman et al., 2009). Raw data examining each estrous cycle phase separately can be found in Supplementary Figures 2–5. Two-way ANOVAs followed by Tukey's *post-hoc* tests were conducted to examine the relationship between gonadal hormonal status (male vs. diestrus/estrus female vs. proestrus female rats) and stress (control vs. stress) for each task during the testing phase. In both cases, the same measurements were collected as during the training phase, plus Omissions for Side Discrimination and Side Reversal, as well as Perseverative Errors and Regressive Errors for Side Reversal and Light Discrimination. Two-way ANOVAs followed by Tukey's *post-hoc* tests were also carried out to examine the relationship between sex (male vs. female rats) and stress (control vs. stress) on *c-fos* expression in the IL, PrL, and OFC subregions of the PFC and on orexin-expressing cells in the lateral hypothalamus. Additionally, two-way ANOVAs followed by Tukey's *post-hoc* tests were performed to examine the relationship between gonadal hormonal status (male vs. diestrus/estrus female vs. proestrus female rats) and stress (control vs. stress) on orexin-expressing cells in the lateral hypothalamus. Lastly, correlations were performed to determine the relationship between *c-fos* expression or orexin expression and task performance during the cognitive flexibility testing phase. The level of significance for all the analyses was set at *p* < 0.05.

## Results

### Sex differences in the training phase of the operant strategy-shifting paradigm

**Figure 2:**
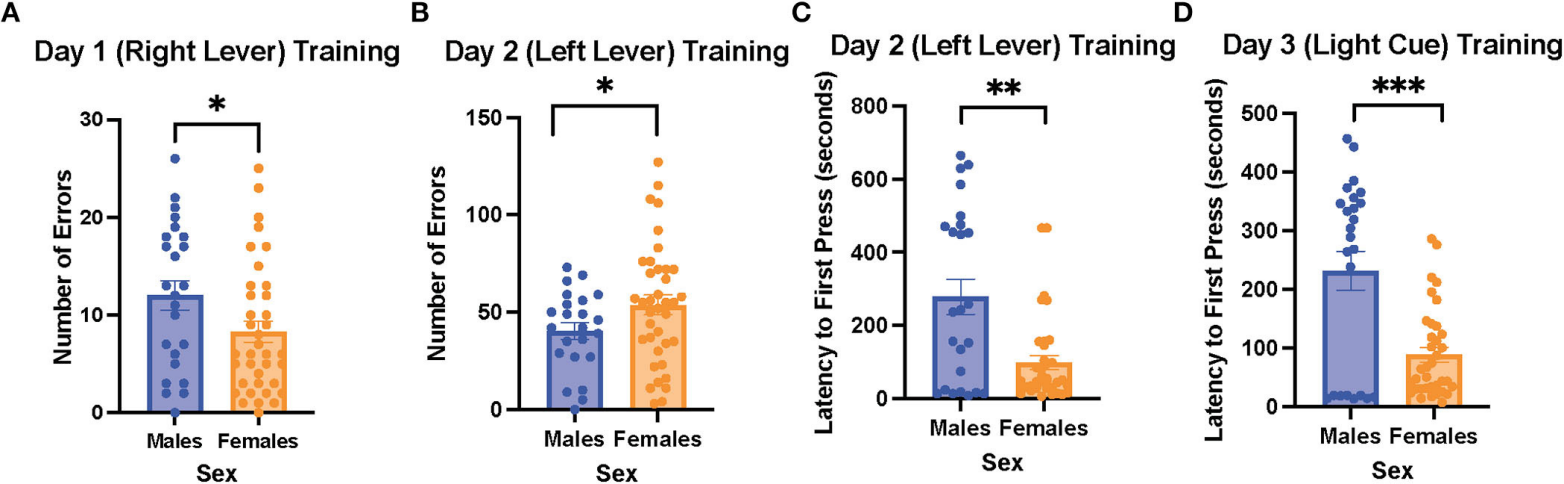
Female rats made fewer errors than male rats when learning to press the right lever on the 1st day of training, but they made more errors than the male rats when learning to press the left lever on the 2nd day of the training; the female rats also showed a shorter latency to first press than the male rats when learning to press the left lever and respond to the light cue. **(A)** Number of errors on the 1st day of the training (learning to press the right lever) in the males and female rats. **(B)** Number of errors on the 2nd day of the training (learning to press the left lever) in the male and female rats. **(C)** Latency to first press on the 2nd day of the training (learning to press the left lever) in the male and female rats. **(D)** Latency to first press on the 3rd day of the training (learning to respond to the light cue) in the male and female rats. Independent samples *t*-tests were conducted to compare the measurements between the mean of males (*n* = 24) and the mean of females (*n* = 40). Error bars are plotted as mean ± SEM. *p < 0.05, **p < 0.01, and***p < 0.001.

On the 1st day of training for the operant lever pressing task, the female rats made fewer errors than the male rats (learning to press the right lever, male rats = 12 ± 1.5 errors vs. female rats = 8.3 ± 1.1 errors, *t*_(59)_ = 2.031, *p* = 0.047; Figure 2A). However, the female rats made more errors than the male ones on day 2 of the training (learning to press the left lever, male rats = 40.5 ± 4.3 errors vs. female rats = 53.7 ± 0.0 errors, *t*_(58)_ = 2.009, *p* = 0.044; Figure 2B). In addition, the female rats exhibited a shorter latency to first press than the male rats on day 2 of the training (male rats = 278.6 ± 48.3 s vs. female rats = 98.9 ± 19.4 s, *t* (31) = 3.451, *p* < 0.001; Figure 2C). However, there were no sex differences in trials to criterion and time to criterion on the 1st and 2nd days of the training (data not shown). On the 3rd day of the training (learning to respond to the light cue), the female rats again showed a significantly shorter latency to first press compared to the male rats (female rats = 88.5 ± 12.7 vs. male rats = 231.5 ± 33.2 s, *t* (30) = 4.023, *p* < 0.001; Figure 2D), while trials to criterion, number of errors, time to criterion, and number of omissions did not show significant differences between the female and male rats (data not shown). Overall, our results suggest that during the training phase of the cognitive flexibility test, the female rats made fewer errors when learning to press the lever differentiated by its location on day 1 of the training but made more errors than the male rats on day 2 of the training on the opposite lever. Moreover, the female rats demonstrated a shorter latency to first press than the male rats on the 2nd and 3rd days of the training.

### Restraint stress behaviors did not differ between the male and female rats

**Figure 3:**
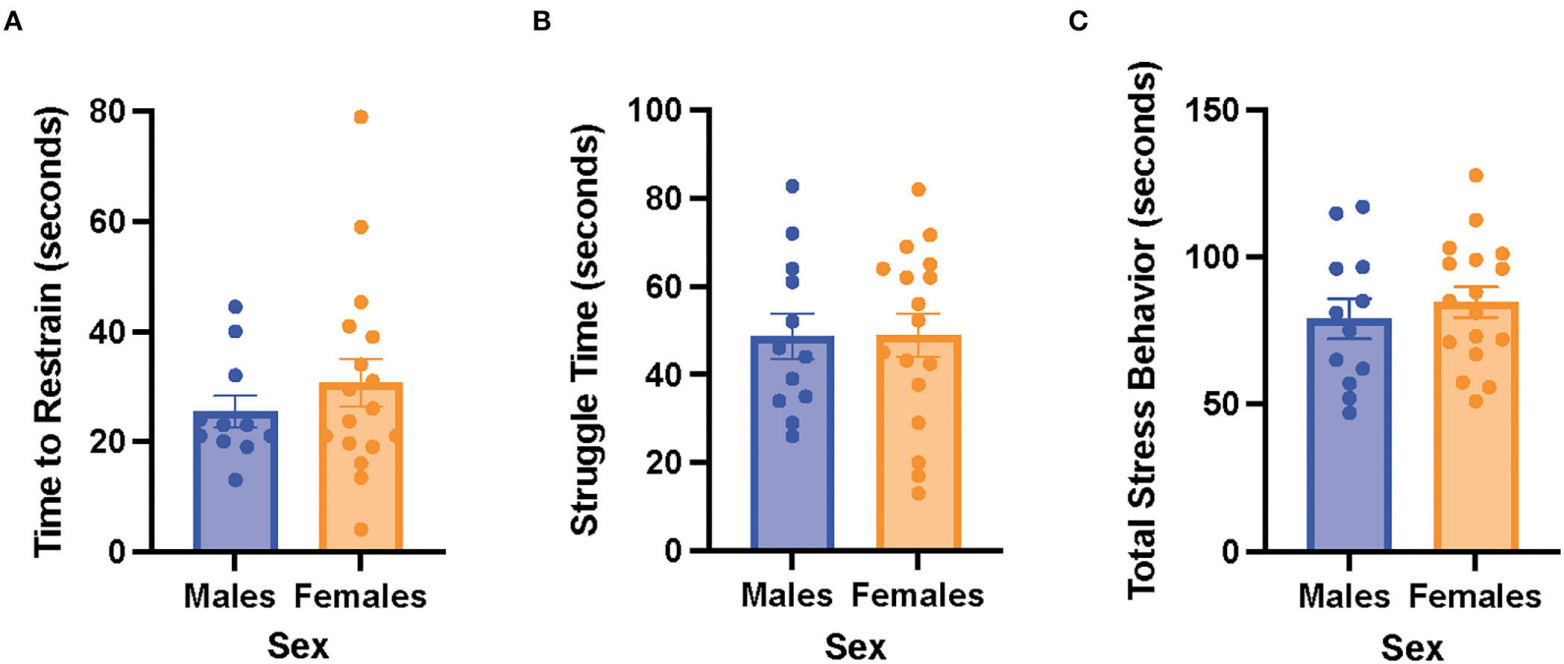
Male and female rats did not differ in restraint stress behaviors. **(A)** Time to restrain in the male and female rats. **(B)** Struggle time in the male and female rats. **(C)** Total stress behavior in the male and female rats. Independent samples *t*-tests were conducted to compare the measurements between the mean of males (*n* = 12) and the mean of females (*n* = 18). Error bars are plotted as mean ± SEM.

There were no significant differences in time to restrain, struggle time, and total stress behavior between the male and female rats (Figure 3). The results suggest that the male and female rats exhibited similar stress behaviors when exposed to acute restraint stress.

### Acute stress effects and sex differences in cognitive flexibility

**Figure 4:**
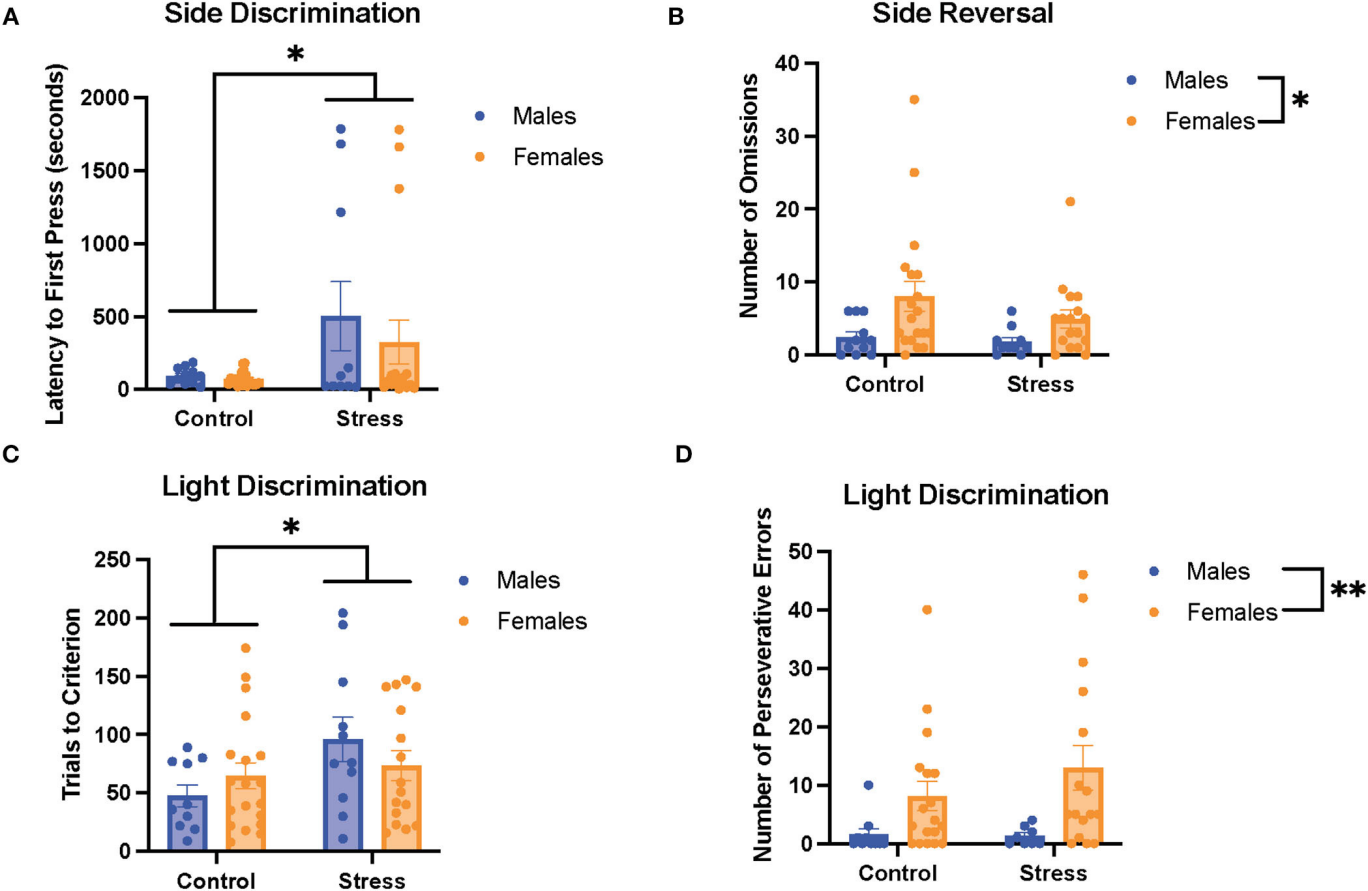
Acute stress and sex impacted performance in the side discrimination, side reversal, and light discrimination tasks. **(A)** Stress increased latency to first press in the side discrimination task in both sexes. **(B)** Female rats exhibited a higher number of omissions than male rats in the side reversal task regardless of stress condition. **(C)** Acute stress increased trials to criterion in the light discrimination task (main effect of stress). **(D)** Female rats demonstrated a higher number of perseverative errors in the light discrimination task compared with male rats. Two-way ANOVAs followed by the Tukey *post-hoc* tests were conducted to examine the relationship between stress and sex, and the interaction between the two variables on the measurements of task performance for the male rats (*n* = 21, control = 11, and stress = 10) and female (*n* = 40: control = 20, and stress = 20) rats. Error bars are plotted as mean ± SEM. *p < 0.05 and ** *p* < 0.01.

We first examined the relationship between stress (control vs. stress) and sex (male vs. female) in each task in the cognitive flexibility test (Figure 4). Stress had a main effect on the latency to first press for the first task of the test [side discrimination, *F*_(1, 53)_ = 6.952, *p* = 0.011, η^2^ = 0.115; Figure 4A, for graph depicting only the main effect of stress, refer to Supplementary Figure 6A] and on trials to criterion for the 3rd task of the test [light discrimination, *F*_(1, 52)_ = 4.286, *p* = 0.043, η^2^ = 0.075; Figure 4C; for graph depicting only the main effect of stress, refer to Supplementary Figure 6C]. Specifically, stress increased the latency to first press in both sexes in the side discrimination task. Moreover, stress increased the trials to criterion in the light discrimination task.

Sex exerted main effects on the number of omissions in the 2nd task [side reversal, *F*_(1, 53)_ = 6.9, *p* = 0.011, η^2^ = 0.11; Figure 4B; for graph depicting only the main effect of sex, refer to Supplementary Figure 6B] and the number of perseverative errors in the 3rd task [light discrimination, *F*_(1, 48)_ = 8.627, *p* = 0.005, η^2^ = 0.148; Figure 4D; for graph depicting only the main effect of sex, refer to Supplementary Figure 6D]. In short, the female rats demonstrated a higher number of omissions and perseverative errors than the male rats in these tasks. There were no significant sex differences or effects of stress for the other measurements of task (data not shown). In sum, the results suggest that stress led to some impairment in task performance by evoking longer latency to first press for the side discrimination task and greater trials to criterion for the light discrimination task (Figures 4A,C). In addition, the data indicate that the female rats showed worse cognitive flexibility performance than the male rats, demonstrated by a higher number of omissions in the side reversal task and more perseverative errors in the light discrimination task (Figures 4B,D).

### The estrous cycle and its interaction with stress to affect cognitive flexibility

**Figure 5:**
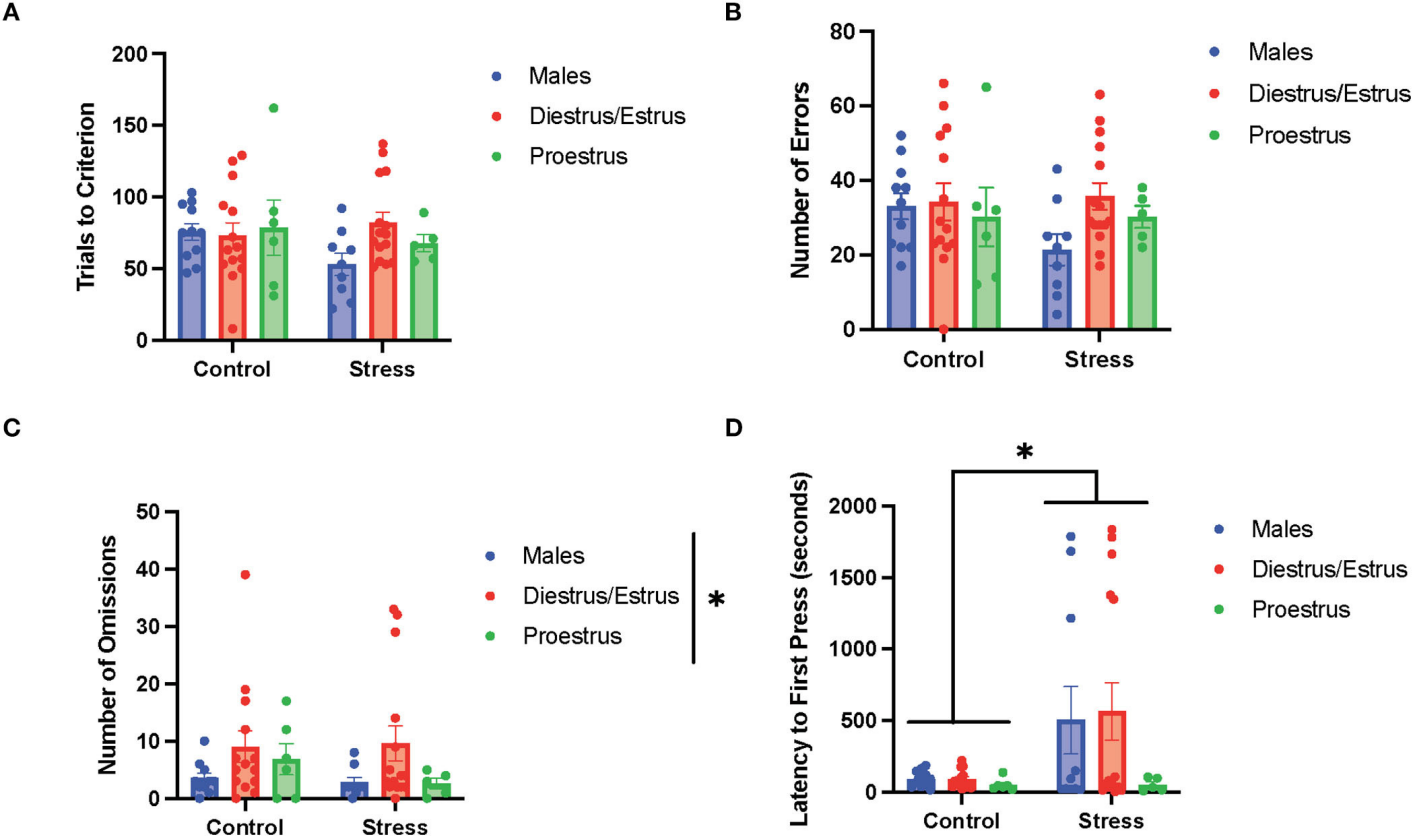
Gonadal hormone status and acute stress impacted performance in the side discrimination task. **(A)** There were no effects of gonadal hormone status or acute stress on trials to criterion. **(B)** There were no effects of gonadal hormone status or acute stress on number of errors. **(C)** There was a main effect of gonadal hormone status on the number of omissions. Although the female rats appear to have higher omissions than the male rats in general, the *post-hoc* tests did not indicate that any particular group was significantly different from another. **(D)** Stress increased the latency to first press for the male and diestrus/estrus female rats. Two-way ANOVAs followed by Tukey *post-hoc* tests were conducted to examine the relationship between stress and gonadal hormone status, and the interaction between the two variables on the measurements of side discrimination task performance for the male (*n* = 21, control = 11, stress = 10) and female (*n* = 40: control diestrus/estrus = 14, control proestrus = 6, stress diestrus/estrus = 14, and stress proestrus = 6) rats. Error bars are plotted as mean ± SEM. *p < 0.05.

**Figure 6:**
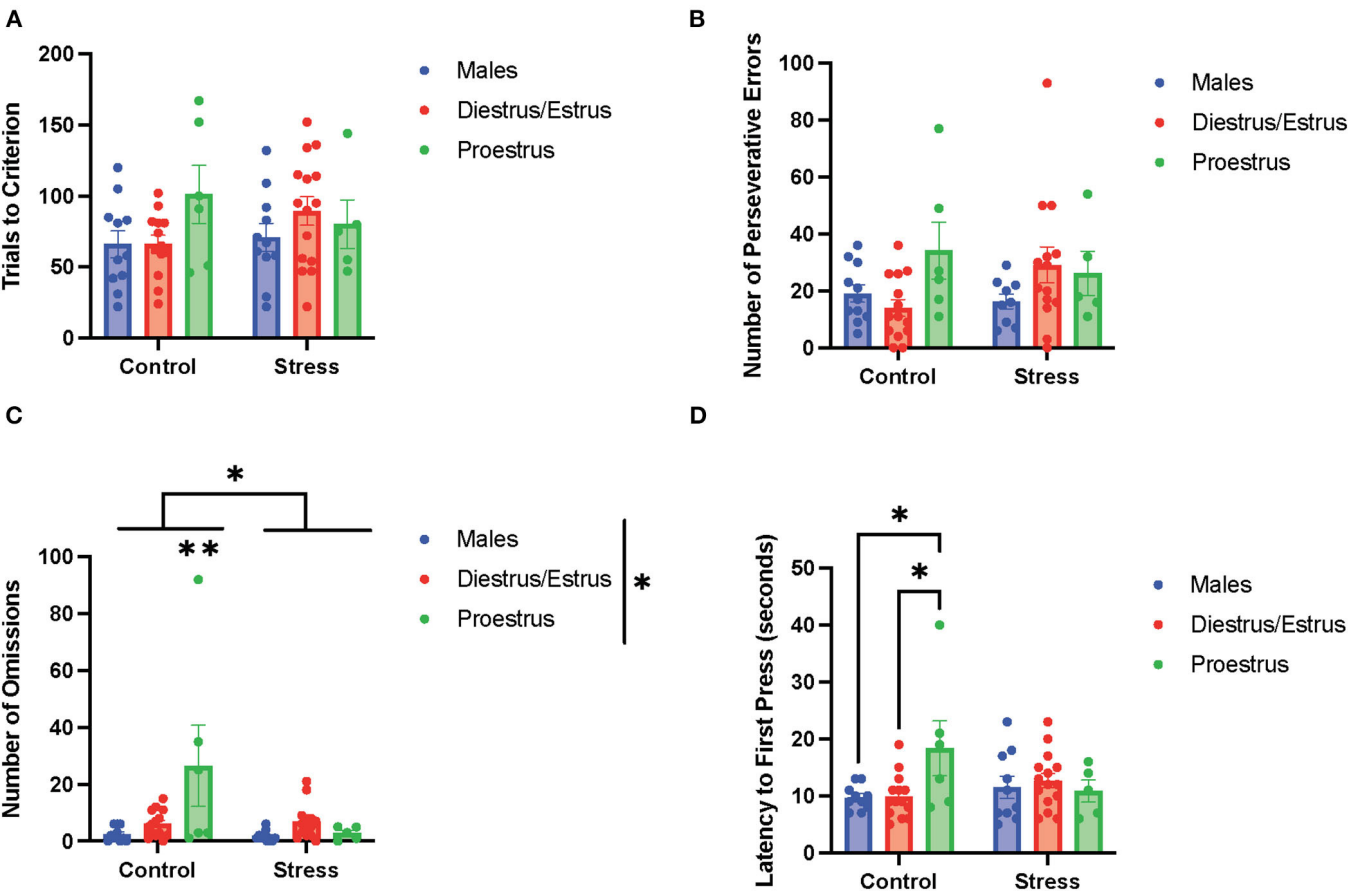
Gonadal hormone status and acute stress impacted performance in the side reversal task. **(A)** Gonadal hormone status and acute stress did not affect the trials to criterion. **(B)** Gonadal hormone status and acute stress did not affect the number of perseverative errors. **(C)** There was a main effect of stress and gonadal hormone status, and an interaction between the two variables on the number of omissions; this was driven by the most omissions by the control proestrus female rats. **(D)** There was an interaction between gonadal hormone status and stress on latency to first press. Specifically, the control proestrus female rats exhibited a higher latency to first press than the control male or control diestrus/estrus female rats. Two-way ANOVAs followed by Tukey *post-hoc* tests were conducted to examine the relationship between stress and gonadal hormone status, and the interaction between the two variables on the measurements of side reversal task performance for the males (*n* = 21: control = 11, and stress = 10) and female (*n* = 40: control diestrus/estrus = 14, control proestrus = 6, stress diestrus/estrus = 14, and stress proestrus = 6) rats. Error bars are plotted as mean ± SEM. *p < 0.05 and **p < 0.01.

**Figure 7:**
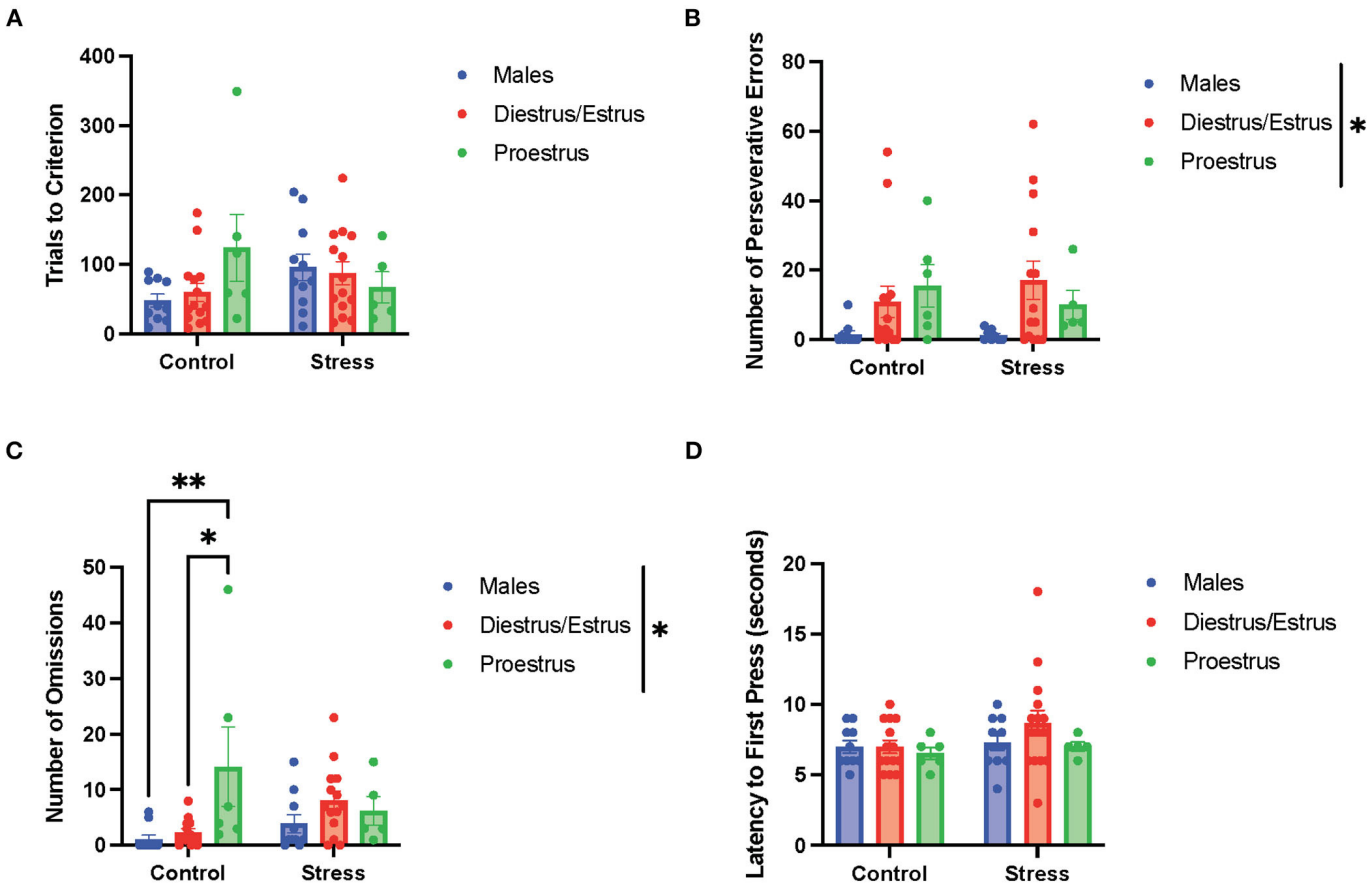
Gonadal hormone status and acute stress impacted performance in the light discrimination task. **(A)** Gonadal hormone status and acute stress did not affect trials to criterion. **(B)** There was a main effect of gonadal hormone status on the number of perseverative errors. Although the female rats appear to have higher perseverative errors than the male rats in general, the *post-hoc* tests did not indicate that any particular group was significantly different from another. **(C)** There was a main effect of gonadal hormone status and an interaction between gonadal hormone status and stress on the number of omissions in the light discrimination task. Moreover, the *post-hoc* tests revealed that the control proestrus female rats had a higher number of omissions than the control male and control diestrus/estrus female rats. **(D)** Gonadal hormone status and acute stress did not affect the latency to first press. Two-way ANOVAs followed by Tukey *post-hoc* tests were conducted to examine the relationship between stress and gonadal hormone status, and the interaction between the two variables on the measurements of light discrimination task performance for the male (*n* = 21: control = 11, and stress = 10) and female (*n* = 40, control diestrus/estrus = 14, control proestrus = 6, stress diestrus/estrus = 14, and stress proestrus = 6). Error bars are plotted as mean ± SEM. *p < 0.05 and **p < 0.01.

To determine the if particular estrous cycle phases drove the sex differences, two-way ANOVAs were conducted to examine the relationship between gonadal hormone status (male vs. diestrus/estrus female vs. proestrus female rats) and stress (control vs. stress) on measurements of performance with each of the three tasks (Figures 5–**7**). In the side discrimination task (the first task during the testing phase, Figure 5), there were no significant main effects of gonadal hormone status or stress on trials to criterion (Figure 5A), number of errors (Figure 5B), or time to criterion (data not shown). However, there was a main effect of gonadal hormone status on the number of omissions [*F*_(2, 53)_ = 3.41, p = 0.041; η^2^ = 0.113; Figure 5C; for graph depicting only the main effect of gonadal hormone status, refer to Supplementary Figure 7A]. Although the female rats appear to have higher omissions than the male rats in general, the *post-hoc* tests did not indicate that any particular group was significantly different from another. In contrast, there was a main effect of stress on latency to first press [*F*_(1, 55)_ = 4.644, *p* = 0.035, η^2^ = *0*.038; Figure 5D; for graph depicting only the main effect of stress, refer to Supplementary Figure 7B]. Specifically, stress increased the latency to first press, and this appeared to be driven by the male and diestrus/estrus female rats in the stress condition. In sum, the data suggest that the female rats made more omissions than the male rats in the side discrimination task, although no particular estrous cycle phase appeared to drive this difference. Moreover, stress led to longer latency to first press in the side discrimination task (Figure 5).

In the side reversal task (the second task during the testing phase; Figure 6), there were no main effects of gonadal hormone status, stress, or interactions between the two variables on trials to criterion (Figure 6A), time to criterion (data not shown), number of errors (data not shown), number of perseverative errors (Figure 6B), and number of regressive errors (data not shown). Interestingly, there were main effects of gonadal hormone status and stress, and an interaction between the two variables on the number of omissions [gonadal hormone status, *F*_(2, 54)_ = 4.34, *p* = 0.018, η^2^ = *0*.114; stress, *F*_(1, 54)_ = 6.193, *p* = 0.011, η^2^ = *0*.081; gonadal hormone status × stress, *F*_(3, 49)_ = 2.81, *p* = 0.049, η^2^ = *0*.128; Figure 6C; for separate graphs depicting the main effects of gonadal hormone status or stress, refer to Supplementary Figures 7C,D, respectively]. The *post-hoc tests* demonstrated significantly more omissions by the control proestrus female rats than the control or stressed male rats, as well as the control or stressed diestrus/estrus female and stressed proestrus female rats (*p* = 0.002,.001,.007,.010, and.013, respectively). Moreover, there was an interaction between gonadal hormone status and stress on latency to first press [*F*_(2, 54)_ = 3.553, *p* = 0.035, η^2^ = 0.108, Figure 6D]. Specifically, the control proestrus female rats exhibited higher latency to first press than the control male or control diestrus/estrus female rats. In sum, female rats in the proestrus phase without exposure to stress exhibited the highest number of omissions and highest latency to first press in the side reversal task.

In the light discrimination task (third task during the testing phase; Figure 7), there were no main effects of gonadal hormone status, stress, or interactions between the two variables on trials to criterion (Figure 7A), number of errors (data not shown), time to criterion (data not shown), number of regressive errors (data not shown), or latency to first press (Figure 7D). The two-way ANOVAs revealed a main effect of gonadal hormone status on perseverative errors [*F*_(2, 51)_ = 4.338, *p* = 0.018, η^2^ = *0*.141; Figure 7B; for graph depicting only the main effect of gonadal hormone status, refer to Supplementary Figure 7E]. Although the female rats appear to have higher perseverative errors than the male rats in general, the *post-hoc* tests did not indicate that any particular group was significantly different from another. Another two-way ANOVA revealed a main effect of gonadal hormone status, as well as an interaction between gonadal hormone status and stress on the number of omissions in the light discrimination task [gonadal hormone status, *F*_(2, 52)_ = 4.277, *p* = 0.019, η^2^ = *0*.125; gonadal hormone status × stress, *F*_(2, 52)_ = 3.79, *p* = 0.029, η^2^ = *0*.107; Figure 7C; for graph depicting the main effect of gonadal hormone status, refer to Supplementary Figure 7F]. Moreover, the *post-hoc* tests revealed that the control proestrus female rats had a higher number of omissions than the control male and control diestrus/estrus female rats (*p* = 0.008 and = 0.0138, respectively; Figure 7C). In summary, the results suggest that the female rats committed more perseverative errors than the male rats, although no particular estrous cycle phase drives this difference (Figure 7B). Additionally, control female rats in the proestrus phase made the highest number of omissions (Figure 7C), but this effect diminished with stress exposure.

### The role of the PFC in sex differences and acute stress effects on cognitive flexibility

**Figure 8:**
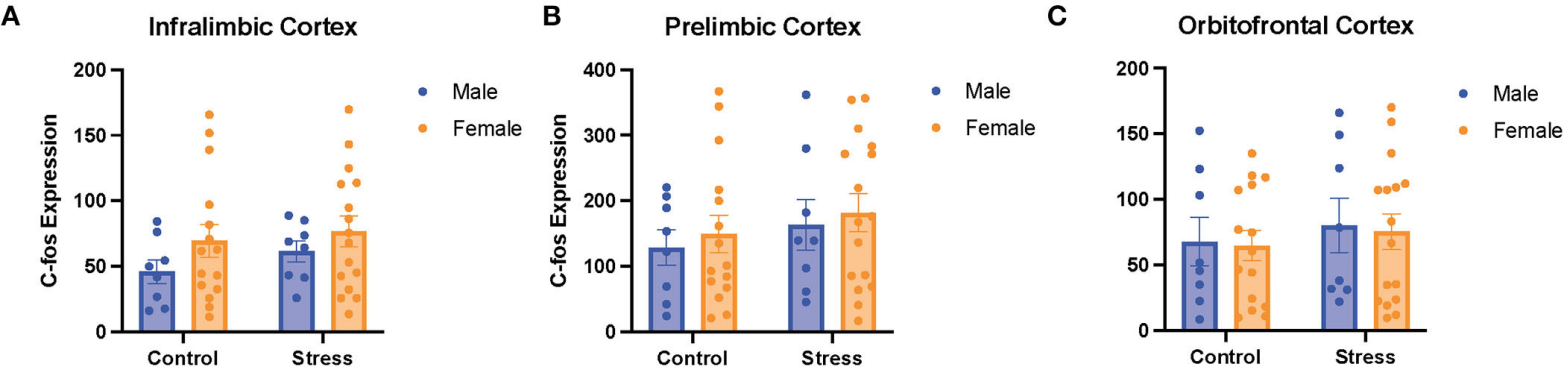
There was no effect of stress or sex on *c-fos* expression in different subregions of the PFC. *C-fos* expression in the **(A)** infralimbic cortex, **(B)** prelimbic cortex, and **(C)** orbitofrontal cortex for the male and female rats in the control or stress conditions. Two-way ANOVAs followed by Tukey *post-hoc* tests were conducted to examine the relationship between stress, sex, and *c-fos* expression for the male (*n* = 16: control = 8, and stress = 8) and female (*n* = 32, control = 16, and stress = 16) rats. Error bars are plotted as mean ± SEM.

A two-way ANOVA was conducted to examine the relationship between sex (male vs. female) and stress (control vs. stress) on *c-fos* expression in the PFC (more specifically in the IL, PrL, and OFC subregions). There were no main effects of sex, stress, or an interaction between sex and stress on *c-fos* expression in the 3quantified subregions of the prefrontal cortex (Figure 8). There were also no significant main effects of gonadal hormone status, stress, or an interaction between the two variables in the three subregions of the PFC (data not shown). However, there were negative correlations between *c-fos* expression in the mPFC (IL and PRL) and omissions in the side reversal and light discrimination tasks, respectively [data not shown; *r*_(37)_ = −0.34, *p* = 0.04; *r*_(38)_ = −0.35, *p* = 0.03]. Specifically, more activation in the mPFC was associated with fewer omissions in those tasks. In sum, the activation in the different subregions of the PFC does not appear to differ between sexes or change significantly with acute stress. However, the activity in the mPFC is correlated with behavioral performance, such that more mPFC activation is associated with fewer omissions.

### The role of orexins in sex differences and acute stress effects on cognitive flexibility

**Figure 9:**
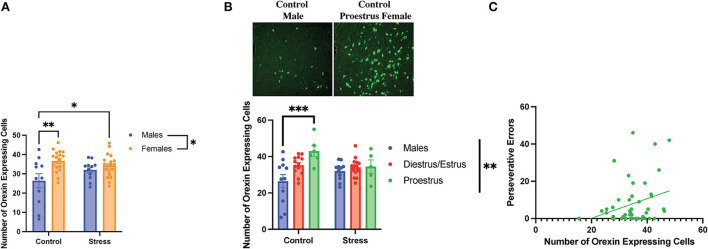
Sex, gonadal hormone status, and stress affected the number of orexin-expressing cells. **(A)** There was a main effect of sex and an interaction between sex and stress on the number of orexin-expressing cells in the lateral hypothalamus. Specifically, the female rats in both the control and stress conditions had a higher number of orexin-expressing cells than the control male rats. **(B)** There was a main effect of gonadal hormone status and an interaction between gonadal hormone status and stress on the number of orexin-expressing cells. Interestingly, the proestrus female rats in the control group had more orexin-expressing cells than the male rats in the control group (representative image above of the number of orexin-expressing cells in the control male rats vs. the control proestrus female rats at Bregma level −3.3 mm). However, stress brought the male and female rats to comparable levels. **(C)** Number of orexin-expressing cells is positively correlated with perseverative errors in the light discrimination (LD) task. Two-way ANOVAs followed by Tukey *post-hoc* tests were conducted to examine the relationship between stress and sex or gonadal hormone status, and the interaction between the two variables on the number of orexin-expressing cells for the male rats. A correlation was performed to determine the relationship between the number of orexin-expressing cells and perseverative errors in the light discrimination task for the male (*n* = 24, control = 12, and stress = 12) and female (*n* = 40: control = 20, stress = 20; control diestrus/estrus = 14, control proestrus = 6, stress diestrus/estrus = 14, and stress proestrus = 6) rats. Error bars are plotted as mean ± SEM. *p < 0.05, **p < 0.01, and ***p < 0.001.

A two-way ANOVA was conducted to examine the relationship between sex (male vs. female) and stress (control vs. stress) on the number of orexin-expressing cells in the lateral hypothalamus. There was a main effect of sex and an interaction between sex and stress on the number of detected orexin-expressing cells [sex, *F*_(1, 55)_ = 10.44, *p* = 0.002, η^2^ = *0*.1498; sex × stress, *F*_(1, 55)_ = 4.281, *p* = 0.043, η^2^ = *0*.061; Figure 9A, for graph depicting only the main effect of sex, refer to Supplementary Figure 8A]. The *post-hoc* test revealed that the female rats in both control and stress conditions had more orexin-expressing cells than the male rats in the control condition (*p* = 0.003 and 0.03 respectively; Figure 9A). In addition, a two-way ANOVA was conducted to examine the relationship between gonadal hormone status (male vs. diestrus/estrus female vs. proestrus female rats) vs. stress (control vs. stress) on the number of orexin-expressing cells in the lateral hypothalamus. Th results revealed a main effect of gonadal hormone status and an interaction between gonadal hormone status and stress on the number of orexin-expressing cells [gonadal hormone status, *F*_(2, 54)_ = 6.812, *p* = 0.002, η^2^ = *0*.181; gonadal hormone status × sex *F*_(2, 54)_ = 3.431, η^2^ = *0*.091; Figure 9B; for graph depicting only the main effect of gonadal hormone status, refer to Supplementary Figure 8B]. The *post-hoc* tests revealed that the control proestrus female rats had more detectable orexin-expressing cells than the control male rats (*p* = 0.001). The data demonstrate that there is a sex difference in the number of orexin-expressing cells in the control condition; namely, that the female rats express more detectable orexin cells than the male rats, and that this is driven by the proestrus female rats. In addition, the data indicate that stress brings the male rats to a similar level of orexin-expressing cells as the female rats in the restraint stress condition. Interestingly, the number of orexin-expressing cells was positively correlated with perseverative errors made in the light discrimination task [*r*_(36)_ = 0.4*, p* = 0.015; Figure 9C]; thus, orexin neurons may be important in set-shifting performance. In sum, when not exposed to stress, the female rats in proestrus exhibit more orexin-expressing cells than the male rats; stress equalizes the number of detectable orexinergic neurons in the male and female rats. Importantly, orexin neurons may play a role in errors made during extradimensional shifts during tests of cognitive flexibility.

## Discussion

Stress is associated with psychiatric disorders such as post-traumatic stress disorder (PTSD) and major depressive disorder (MDD) (Carr et al., 2013; Bangasser and Valentino, 2014), which are characterized in part by cognitive inflexibility (Powell and Ragozzino, 2017; Ben-Zion et al., 2018; Boisseau and Garnaat, 2018; Doss et al., 2021) and are more common in women (Nestler et al., 2002; Keane et al., 2006; Bangasser and Valentino, 2014; Swaab and Bao, 2020). It is important to consider where women are in their cycle when exposed to stress and cognitive flexibility testing, as gonadal hormones can contribute to sex differences in response to stress (Becker et al., 2005; Oyola and Handa, 2017; Heck and Handa, 2019). Although previous studies have separately examined sex differences in cognitive flexibility (LaClair et al., 2019; Hilz et al., 2022), stress effects on cognitive flexibility (Thai et al., 2013; Hurtubise and Howland, 2016; Shields et al., 2016; Goldfarb et al., 2017; Grafe et al., 2017a; Sullivan et al., 2019), and the effect of gonadal hormones on cognitive flexibility (Wallin and Wood, 2015; Lipatova et al., 2016; Hilz et al., 2022), many of the findings were inconsistent, and the role of the estrous cycle in stress-induced impacts on cognitive flexibility is still unknown. This study explored sex differences in cognitive flexibility using an operant strategy-shifting paradigm after either control conditions or restraint stress in freely cycling female and male rats (with estrous cycle tracking in the female rats). In addition, we examined potential neural correlates for any changes in behavior observed not only to build our understanding of biological mechanisms behind cognitive flexibility but also to lead to more targeted treatments for psychiatric disorders associated with impairments of cognitive flexibility.

### Sex differences in the training phase of the operant strategy shifting paradigm

While the female rats initially performed better than the male rats during the 1st day of the training for the operant strategy-shifting paradigm, the female rats perseverated on the incorrect lever on the 2nd day of the training, making more errors than the male rats. Our results are consistent with previous findings in primates that females exhibit worse reversal learning (LaClair et al., 2019). In addition, we did not observe sex differences on day 3 of the training (light cue, a new dimension), which is consistent with previous findings in primates that females and males require comparable trials to criterion for extradimensional shift learning (LaClair et al., 2019). The better learning observed in the female rats on the first day might be because they are more sensitive to the food reward following dietary restriction, leading to better initial stimulus response learning with the food reward as response. Indeed, previous studies have shown that food reward may be stronger in female rats than in male rats (Sinclair et al., 2017). Interestingly, we also found that the female rats pressed the lever more quickly than the male ones on the 2nd and 3rd days of the training. Considering that shorter latency to first press has been interpreted as motivation for food reward in previous studies (Workman et al., 2013), our results suggest that the female rats might be more motivated to obtain the food reward during the training, which can also potentially be explained by the greater perception of food reward by female rats (Sinclair et al., 2017).

### Acute stress effects on cognitive flexibility

We found that acute restraint stress impaired task performance on test day, demonstrated by longer latency to first press (side discrimination task) and more trials to criterion (light discrimination task). The longer latency to first press may suggest that acute stress reduces the motivation for food reward, consistent with results from a previous study exposing rodents to repeated stress (Sullivan et al., 2019). However, our finding that acute stress led to impaired performance on the light discrimination task is not consistent with a previous study examining acute stress in male rats (Thai et al., 2013). Importantly, the operant strategy-shifting paradigm used in this previous study was different from the current study; each task was completed on different days, with either control or stress groups prior to set shifting and reversal (Thai et al., 2013). Moreover, the stress impairments during the light discrimination task in our study were consistent with studies that exposed animals to repeated stress or chronic unpredictable stress (Hurtubise and Howland, 2016). Thus, it appears that acute stress is sufficient to impair adaptive response strategies in a new stimulus dimension, which requires high-level cognitive processes (Brady and Floresco, 2015; Brown and Tait, 2016). Lastly, we did not observe any improvements in reversal learning following 30-min acute restraint stress in the male rats as previously reported, but again, a different operant strategy-shifting paradigm was used (Thai et al., 2013). Interestingly, we did observe that acute stress reduced the number of omissions for the proestrus female rats. This suggests that acute stress improves adaptive response strategies for simpler tasks in stimulus dimensions (Buss, 1956; Harrow and Friedman, 1958).

### Sex differences in cognitive flexibility

We found that the female rats had worse performances than the male rats in both the side reversal and light discrimination tasks on test day, demonstrated by more omissions and perseverative errors, respectively. The impaired side reversal performance in the female rats on test day is parallel with the poor performance by the female rats during the 2nd day of the training (where they had to press the opposite lever from the 1st day of training). However, the impaired performance in the side reversal task on test day is mostly due to omissions, which are failures to respond to a cue, indicating slower cortical processing and attentional deficits (Korgaonkar et al., 2021). Thus, it appears that the female rats demonstrate slower cortical processing than the male rats when trying to adapt to new rules in a stimulus dimension (Brady and Floresco, 2015). In contrast, in the light discrimination task, when a new dimension was involved, the female rats made more perseverative errors than the male rats. This indicates that the female rats might be less likely to inhibit strategies involving previously related dimension in order to explore new strategies. These results indicate that female rats may be more subject to perseveration than male rats, which is defined as the inability to abandon an established strategy for a new strategy despite the fact that the old strategy is no longer useful (Landry and Mitchell, 2021). These errors demonstrate cognitive rigidity and inability to adapt to change (Uddo et al., 1993; Vasterling et al., 1998; Van Laethem et al., 2016; Miles et al., 2021). This sex difference in cognitive flexibility can be explained by the previous observation that female rats form habitual behaviors more quickly or that they are more committed to habitual behaviors than male rats (LaClair et al., 2019). However, it has also been proposed that this sex difference in habitual behaviors is dependent on estrogen levels (LaClair et al., 2019). Therefore, gonadal hormone changes during the estrous cycle likely influence female cognitive flexibility, as discussed below.

### The estrous cycle and its interaction with stress to affect cognitive flexibility

Our results demonstrate that the control proestrus female rats drove most of the sex differences that we observed in cognitive flexibility. Specifically, the proestrus female rats exhibited worse performance in the side reversal and light discrimination tasks than all the other groups primarily because of more omissions and perseverative errors. We will first discuss the reversal performance of the proestrus female rats, followed by their set shifting performance and, lastly, we will consider the effects that stress had on the proestrus female rats in these tasks.

We expected a poor reversal performance by the proestrus female rats, as estradiol levels are highest during proestrus compared to the other phases of the estrous cycle (Becker et al., 2005; Heck and Handa, 2019), and estradiol treatment causes impairments in reversal learning in marmosets (Lacreuse et al., 2014). However, it is important to consider that progesterone levels are also high in the proestrus phase (Heck and Handa, 2019). Other studies have indicated that performance in reversal learning is best when estradiol levels are high and progesterone levels are low (Kromrey et al., 2015). However, there is no consensus on whether progesterone shows positive or detrimental effects on cognition (Barros et al., 2015). In short, the increased omissions during reversal in the proestrus female rats indicate that high levels of estrogen or progesterone can slow cortical processing while adapting to new rules in a stimulus dimension (Korgaonkar et al., 2021).

We also expected a poor performance in the light discrimination task by the control proestrus female rats, as a previous study demonstrated impaired set-shifting performance in ovariectomized female rats with 17β-estradiol treatment (Hilz et al., 2022). It is important to note that our results contradict two other studies, which are carried out on female rhesus monkeys and female rats, respectively, and showed that estradiol treatment improves set shifting (Voytko et al., 2009; Lipatova et al., 2016). However, as the first study was performed on menopausal monkeys and the rodent study used a different paradigm to assess extradimensional shift, it is possible the research model or paradigm used could explain the conflicting results. It is equally important to consider how progesterone levels during the proestrus phase may affect set shifting. Other studies found that administration of progestin during development increases omissions and perseverative errors in cognitive tasks in adulthood (Willing and Wagner, 2016; Fahrenkopf et al., 2021). The observed omissions and perseverative errors made by the proestrus female rats during the light discrimination task indicate both slower cortical processing and cognitive rigidity (Uddo et al., 1993; Vasterling et al., 1998; Van Laethem et al., 2016; Miles et al., 2021). More research studies will have to be conducted to determine if estrogen, progesterone, or both, contribute to these impairments and the mechanism by which this occurs.

We expected that stress would further exacerbate the cognitive flexibility impairments in the proestrus female rats, as high estrogen and progesterone levels are associated with higher HPA response (Oyola and Handa, 2017; Heck and Handa, 2019). On the contrary, our results indicate that stress abolished the impairments in cognitive flexibility in the proestrus female rats by reducing omissions. This phenomenon might be due to the higher vigilance, cue sensitivity, and enhanced associative learning induced by acute stress (Stelly et al., 2020). Since omissions in cognitive flexibility tasks can reflect attention and vigilance (Vasterling et al., 1998), the decrease in omissions of proestrus female rats exposed to acute stress can demonstrate a higher level of vigilance or sensitivity to cues. In sum, these results imply that acute stress can exert a positive effect on cognitive flexibility in female rats with high estrogen and progesterone levels (proestrus female rats), potentially by increasing vigilance specifically for tasks involving higher level of difficulty or susceptibility to perseveration.

### The role of the PFC in sex differences and acute stress effects on cognitive flexibility

We found that there were no main effects of sex, estrous phase, stress, or interactions between these variables on *c-fos* expression in the 3 quantified subregions of the prefrontal cortex (IL, PrL, and OFC). As previous literature indicates that the PFC is important for cognitive flexibility tasks (McAlonan and Brown, 2003; Placek et al., 2013; Lipatova et al., 2016), we expected to find that the control proestrus female rats would show changes in their PFC activation to reflect impairments in their behavioral performance. However, as previously mentioned, we did not find group differences. Interestingly, we did find negative correlations between *c-fos* expression in the mPFC and omissions in the side reversal and light discrimination tasks. Thus, it appears that activity in the mPFC is correlated with behavioral performance, such that more mPFC activation is associated with fewer omissions. As previously mentioned, omissions in attention tasks appear to be more common in patients with PTSD, indicating slower cortical processing (Vasterling et al., 1998). Thus, it makes sense that more PFC activity is associated with fewer omissions in our task.

Our findings that stress did not significantly alter *c-fos* expression in the PFC is not consistent with previous findings that stress can impair functions of PFC (Arnsten, 2009). However, many of the previous studies examined the effects of chronic, rather than acute, stress. Given that acute stress affected cognitive flexibility performance, our results suggest that acute stress may exert some effects on cognitive flexibility without changing PFC neural activation.

### Orexin neurons play a role in cognitive flexibility

We found that the control female rats had a higher number of detectable orexin-expressing cells than the control male rats. However, acute stress brought both sexes to a similar level of orexin- expressing cells. A previous study has found that acute restraint stress can induce increases in activities of orexin neurons (Grafe et al., 2017b; Grafe and Bhatnagar, 2018), which may explain the higher number of detectable orexin-expressing cells in response to stress observed in our study. In addition, there may be a ceiling effect of orexin production (limited by quantities or rates of production), such that stress cannot increase the number of detectable orexin cells in female rats beyond their already high control levels.

We also found that the control proestrus female rats showed higher orexin expression than the control male rats, which is consistent with previous findings (Porkka-Heiskanen et al., 2004). Interestingly, the control proestrus female rats also showed worse performance in the side reversal and light discrimination tasks than the control male rats. This may suggest that high orexin expression can interfere with reversal learning and extradimensional shifting. In support of this, we found that the number of orexin-expressing cells was positively correlated with the number of perseverative errors in the light discrimination task. In sum, this suggests that high levels of orexin expression may underlie perseveration.

### Limitations

One limitation of our study is that it lacked direct measurements of levels of gonadal hormones such as estrogen and progesterone. In our study, the effects of estrogen and progesterone were extrapolated from levels of the sex steroids associated with each estrous phase based on previous studies (Becker et al., 2005). However, more direct measurements of estrogen levels would be beneficial for more straightforward investigations of the effects of estrogen on task performances. In addition, as testosterone has been also shown to impact performance in cognitive flexibility tasks (Wallin and Wood, 2015; Tomm et al., 2022), and testosterone levels can vary by individuals (Viau, 2002), measurements or experimental designs with testosterone could add more insights into the understanding of sex differences in cognitive flexibility (Shansky, 2019).

Another limitation of our study was the inability to measure the perception of stress in rats, which is a limitation of animal research in general. Differences in stress perception could possibly explain the opposite effects of acute stress on cognitive flexibility in different estrous phases. In rats, cortisol levels can potentially reflect stress perception (Goldfarb et al., 2017; Gabrys et al., 2019). However, the cutoff level of cortisol corresponding to the perception of controllability and uncontrollability can be difficult to determine. In addition, since sympathetic response has also been shown to affect cognitive flexibility (Lapiz and Morilak, 2006; Tait et al., 2007; Hurtubise and Howland, 2016), measurements of sympathetic responses such as noradrenaline level would be important for understanding the effects of acute stress on cognitive flexibility.

Finally, we did not counterbalance the order of cues (i.e., lever location vs. light cue) in our attentional set shifting paradigm. Some research using the pot digging version of the attentional set shifting paradigm suggests that counterbalancing cues is important. Specifically, researchers have found that there are some differences in the simple discrimination stage depending on which cue is introduced first (i.e., higher number of trials to criterion if the odor is the relevant cue), which may then affect subsequent performance (Heisler et al., 2015). Although our operant lever pressing version of the task does not include odor or media cues, it may still be important to counterbalance the lever location cue vs. the light cue in future studies. Importantly, in the lever location dimension, we did calculate side bias during the training and accounted for any observed bias on test day.

## Conclusions and future directions

Our findings provide an insight into how the estrous cycle impacts cognitive flexibility and, moreover, how stress and the estrous cycle interact to affect cognitive flexibility performance in rats. Future studies should investigate whether the positive effects of stress on female rats in the proestrus phase are only limited to acute stress or if chronic stress would exert similar effects. Moreover, future studies should determine how the gonadal hormone milieu affects learning this cognitive flexibility task (during training). In addition, more research is needed to better understand how orexin activity in the lateral hypothalamus underlies cognitive flexibility performance. Indeed, understanding the neural circuits of cognitive flexibility would provide more clarity as to how sex differences in behavior are mediated. Previous research has indicated the importance of dopaminergic input on cognitive flexibility (Floresco et al., 2008; Haluk and Floresco, 2009; Klanker et al., 2013; Radke et al., 2019; Tomm et al., 2022); as orexin neurons project to the VTA (Peyron et al., 1998), understanding how these neural substrates interact could provide more clarity on sex differences in cognitive flexibility after stress. Furthermore, future research should more directly determine the role of gonadal hormones in these circuits, which would lead to a more comprehensive understanding of sex differences in cognitive flexibility. Lastly, future studies should incorporate objective measures of the stress response, including cortisol and noradrenaline levels, to examine the relationship between gonadal hormones, stress response and perception, and cognitive flexibility performance.

Our study contributes to the understanding of sex differences and impacts of stress on cognitive flexibility, as well as the role of the estrous cycle in cognitive flexibility. Since impairments of cognitive flexibility are related to the stress-related psychiatric disorders, which are more prevalent in women than men, unveiling the sex differences and effects of hormone status on cognitive flexibility is crucial to the improvement of diagnosis and treatment of these stress-related psychiatric disorders. Therefore, the findings from our study could potentially inform the development of individualized treatment for psychiatric disorders associated with impaired cognitive flexibility.

## Data availability statement

The raw data supporting the conclusions of this article will be made available by the authors, without undue reservation.

## Ethics statement

The animal study was reviewed and approved by Bryn Mawr Institutional Animal Care and Use Committee.

## Author contributions

AG and LG contributed to the conception and design of the study. AG, JH, IR, AH, XL, KS, and LG performed the experiments, collected the data for the manuscript, and performed the data analysis. JH wrote the first draft of the manuscript. JH, AG, and LG wrote sections of the manuscript. All authors contributed to revision of the manuscript, read, and approved the submitted version.

## Funding

This work was supported by Bryn Mawr College research funds.

## Conflict of interest

The authors declare that the research was conducted in the absence of any commercial or financial relationships that could be construed as a potential conflict of interest.

## Publisher's note

All claims expressed in this article are solely those of the authors and do not necessarily represent those of their affiliated organizations, or those of the publisher, the editors and the reviewers. Any product that may be evaluated in this article, or claim that may be made by its manufacturer, is not guaranteed or endorsed by the publisher.

## Supplementary material

Supplementary Figure 1 Representative vaginal lavage pictures categorized into each stage of the estrous cycle. **(A)** Samples that displayed predominantly leukocytes (and some larger round cells without nuclei) were categorized as diestrus. **(B)** Samples that had primarily nucleated epithelial cells were categorized as proestrus. **(C)**Samples that principally included cornified cells were categorized as estrus.

Supplementary Figure 2 Gonadal hormone status and acute stress impacted performance in the side discrimination task. **(A)** There was a main effect of gonadal hormone status on the trials to criterion, with the diestrus female rats showing the highest trials to criterion regardless of stress. **(B)** There was a main effect of gonadal hormone status on the number of errors, with the diestrus female rats showing the highest number of errors regardless of stress condition. **(C)** There was no effect of stress or gonadal hormone status on the number of omissions. **(D)** Stress increased the latency to first press for the male and diestrus female rats. Two-way ANOVAs followed by Tukey *post-hoc* tests were conducted to examine the relationship between stress and gonadal hormone status, and the interaction between the two variables, on the measurements of side reversal task performance for the male (*n* = 21: control = 11, and stress = 10) and female (*n* = 40, control diestrus = 6, control proestrus = 6, control estrus = 8, stress diestrus = 8, stress proestrus = 6, and stress estrus = 6) rats. Error bars are plotted as mean ± SEM. ^*^*p* < 0.05.

Supplementary Figure 3 Gonadal hormone status and acute stress impacted performance in the side reversal task. **(A)** Gonadal hormone status and acute stress did not affect the trials to criterion. **(B)** Gonadal hormone status and acute stress did not affect the number of perseverative errors. **(C)** There was a main effect of stress and gonadal hormone status, and an interaction between the two variables, on the number of omissions; this was driven by the most omissions of the control proestrus female rats. **(D)** Gonadal hormone status and acute stress did not affect the latency to first press. Two-way ANOVAs followed by Tukey *post-hoc* tests were conducted to examine the relationship between stress and gonadal hormone status, and the interaction between the two variables, on the measurements of side reversal task performance for the male (*n* = 22: control = 11, and stress = 11) and female (*n* = 40, control diestrus = 6, control proestrus = 6, control estrus = 8, stress diestrus = 8, stress proestrus = 6, and stress estrus = 6). Error bars are plotted as mean ± SEM. ^*^*p* < 0.05 and ^**^p < 0.01.

Supplementary Figure 4 Gonadal hormone status and acute stress impacted performance in the light discrimination task. **(A)** Gonadal hormone status and acute stress did not affect trials to criterion. **(B)** There was a main effect of gonadal hormone status on the number of perseverative errors; stressed female rats in the estrus phase made the highest number of errors. **(C)** There was a main effect of gonadal hormone status on the number of omissions; the control proestrus female rats had the highest number of omissions. **(D)** Stress increased the latency to first press. Two-way ANOVAs followed by Tukey *post-hoc* tests were conducted to examine the relationship between stress and gonadal hormone status, and the interaction between the two variables, on the measurements of light discrimination task performance for male (*n* = 22: control = 11, and stress = 11) and female (*n* = 40, control diestrus = 6; control proestrus = 6; control estrus = 8, stress diestrus = 8, stress proestrus = 6, and stress estrus = 6). Error bars are plotted as mean ± SEM. ^*^*p* < 0.05.

Supplementary Figure 5 Sex, gonadal hormone status, and stress affected the number of orexin-expressing cells. **(A)** There was a main effect of sex, and an interaction between sex and stress, on the number of orexin-expressing cells in the lateral hypothalamus. Specifically, the female rats in both the control and stress conditions had a higher number of orexin-expressing cells than the control male rats. **(B)** There was a main effect of gonadal hormone status, and an interaction between gonadal hormone status and stress, on the number of orexin-expressing cells; the proestrus female rats in the control group had more orexin-expressing cells than the male rats in the control group (representative image above of the number of orexin-expressing cells in the control male rats vs. the control proestrus female rats at Bregma level−3.3 mm). However, stress brought the male and female rats to comparable levels. **(C)** Number of orexin-expressing cells is positively correlated with perseverative errors in the light discrimination (LD) task. Two-way ANOVAs followed by Tukey *post-hoc* tests were conducted to examine the relationship between stress and sex or gonadal hormone status, and the interaction between the two variables, on the number of orexin-expressing cells for the males (*n* = 24, control = 12 and stress = 12) and female (*n* = 40: control = 20; stress = 20; control diestrus = 6; control proestrus = 6; control estrus = 8; stress diestrus = 8; stress proestrus = 6, and stress estrus = 6). A correlation was performed to determine the relationship between the number of orexin-expressing cells and perseverative errors in the light discrimination task. Error bars are plotted as mean ± SEM. ^*^*p* < 0.05 and ^**^*p* < 0.01.

Supplementary Figure 6 Main effects of stress or sex displayed in graph format for original Figure 4 data. Acute stress and sex impacted performance in the side discrimination, side reversal, and light discrimination tasks. **(A)** Stress increased latency to first press in the side discrimination task. **(B)** Female rats exhibited a higher number of omissions than male rats in the side reversal task. **(C)** Acute stress increased trials to criterion in the light discrimination task. **(D)** Female rats demonstrated a higher number of perseverative errors in the light discrimination task than the male rats (*n* = 31 control, 30 stress; *n* = 21 male and 40 female rats).

Supplementary Figure 7 Main effects of stress or gonadal hormone status displayed in graph format for original Figures 5–7 data. **(A)** Gonadal hormone status had a main effect on the number of omissions in the side discrimination task. **(B)** Stress increased the latency to first press in the side discrimination task. **(C)** Gonadal hormone status had a main effect on the number of omissions in the side reversal task. **(D)** Control animals demonstrated more omissions than stressed animals in the side reversal task. **(E)** Gonadal hormone status had a main effect on the number of perseverative errors in the light discrimination task. **(F)** Gonadal hormone status had a main effect on the number of omissions in the light discrimination task. (*n* = 31 control, 30 stress; *n* = 21 male, 28 diestrus/estrus female, and 12 proestrus female rats).

Supplementary Figure 8 Main effects of stress, sex, or gonadal hormone status displayed in graph format for original Figure 9 data. **(A)** Female rats have a higher number of orexin-expressing cells than male rats. **(B)** Gonadal hormone status has a main effect on the number of orexin-expressing cells in the lateral hypothalamus. (*n* = 24 male and 40 female rats; *n* = 24 male, 28 diestrus/estrus female, and 12 proestrus female rats).
