# Supplementary figures and images for: Sex differences in cognitive flexibility are driven by the estrous cycle and stress-dependent
Source: Front Behav Neurosci. 2022 Aug 4;16:958301. doi: 10.3389/fnbeh.2022.958301 (PMC9386277; doi:10.3389/fnbeh.2022.958301)

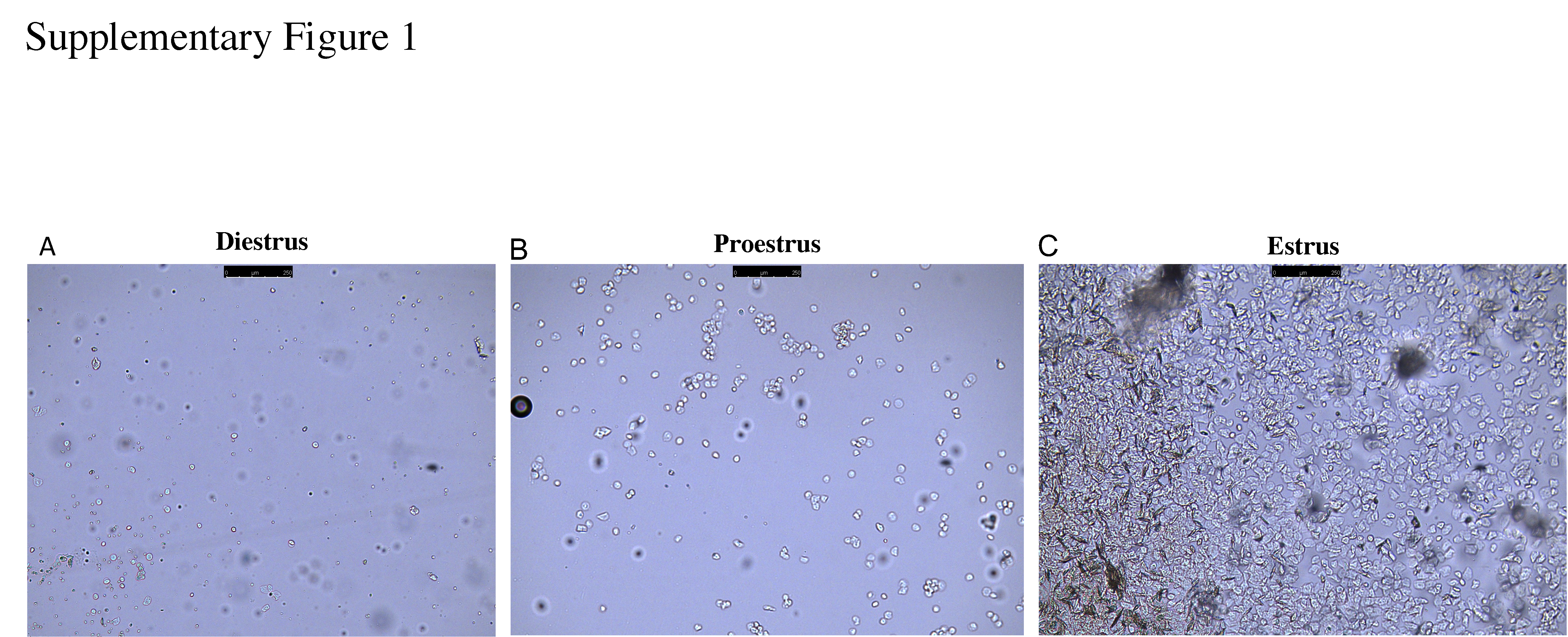

Supplement: Supplementary Figure 1 — Representative vaginal lavage pictures categorized into each stage of the estrous cycle. (A) Samples that displayed predominantly leukocytes (and some larger round cells without nuclei) were categorized as diestrus. (B) Samples that had primarily nucleated epithelial cells were categorized as proestrus. (C)Samples that principally included cornified cells were categorized as estrus. [file Image_1.tiff]

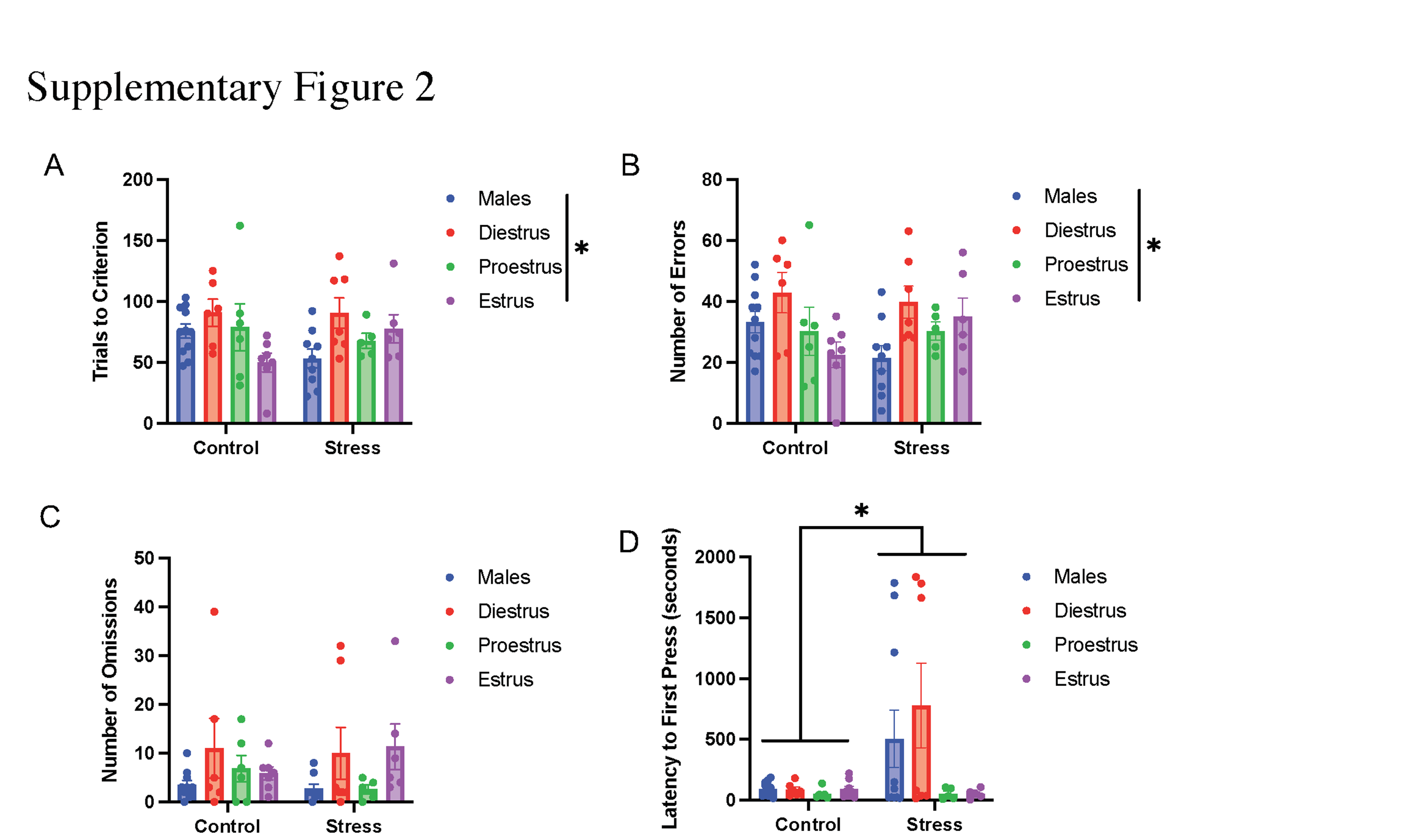

Supplement: Supplementary Figure 2 — Gonadal hormone status and acute stress impacted performance in the side discrimination task. (A) There was a main effect of gonadal hormone status on the trials to criterion, with the diestrus female rats showing the highest trials to criterion regardless of stress. (B) There was a main effect of gonadal hormone status on the number of errors, with the diestrus female rats showing the highest number of errors regardless of stress condition. (C) There was no effect of stress or gonadal hormone status on the number of omissions. (D) Stress increased the latency to first press for the male and diestrus female rats. Two-way ANOVAs followed by Tukey post-hoc tests were conducted to examine the relationship between stress and gonadal hormone status, and the interaction between the two variables, on the measurements of side reversal task performance for the male (n = 21: control = 11, and stress = 10) and female (n = 40, control diestrus = 6, control proestrus = 6, control estrus = 8, stress diestrus = 8, stress proestrus = 6, and stress estrus = 6) rats. Error bars are plotted as mean ± SEM. *p < 0.05. [file Image_2.tiff]

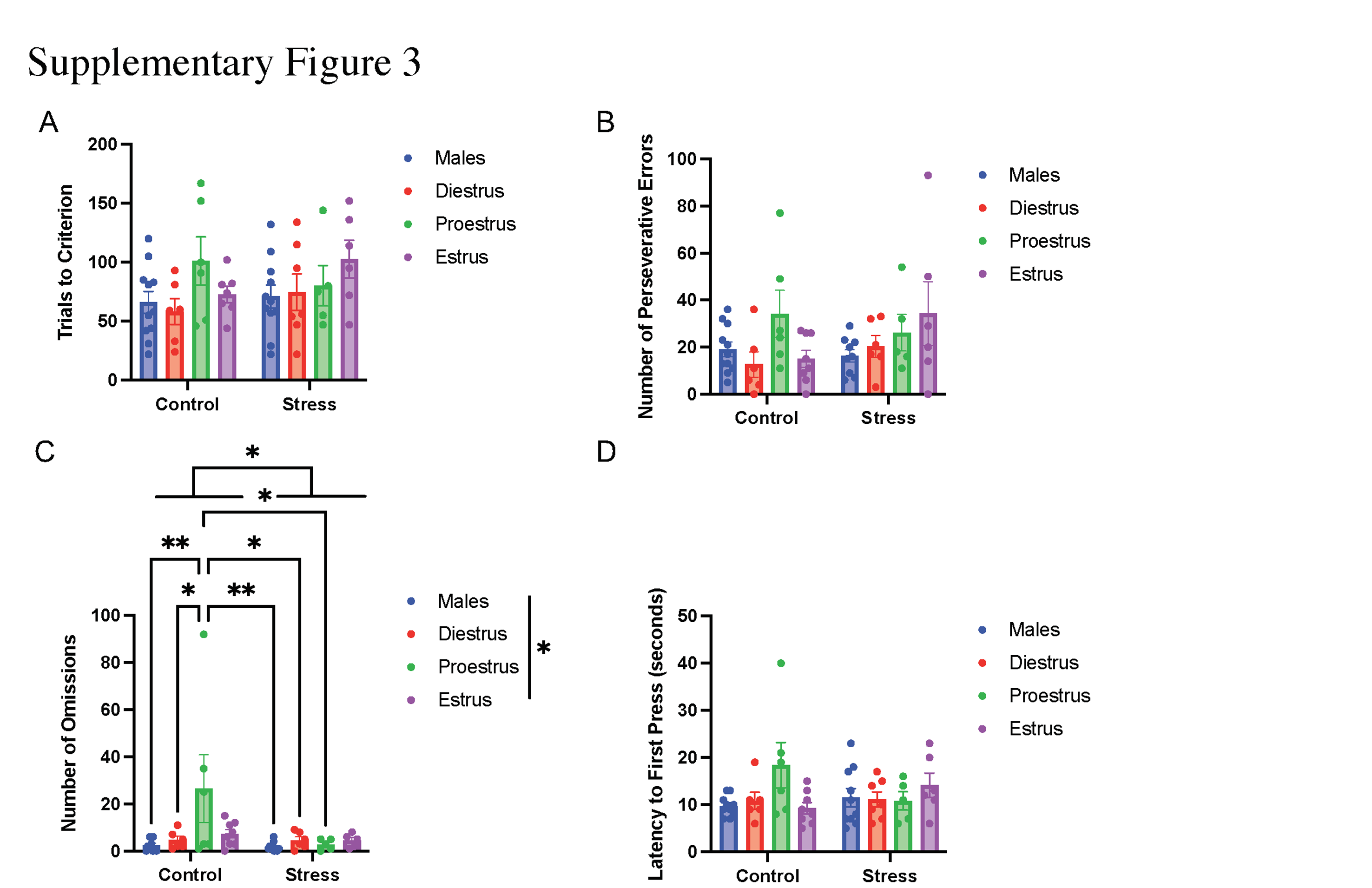

Supplement: Supplementary Figure 3 — Gonadal hormone status and acute stress impacted performance in the side reversal task. (A) Gonadal hormone status and acute stress did not affect the trials to criterion. (B) Gonadal hormone status and acute stress did not affect the number of perseverative errors. (C) There was a main effect of stress and gonadal hormone status, and an interaction between the two variables, on the number of omissions; this was driven by the most omissions of the control proestrus female rats. (D) Gonadal hormone status and acute stress did not affect the latency to first press. Two-way ANOVAs followed by Tukey post-hoc tests were conducted to examine the relationship between stress and gonadal hormone status, and the interaction between the two variables, on the measurements of side reversal task performance for the male (n = 22: control = 11, and stress = 11) and female (n = 40, control diestrus = 6, control proestrus = 6, control estrus = 8, stress diestrus = 8, stress proestrus = 6, and stress estrus = 6). Error bars are plotted as mean ± SEM. *p < 0.05 and **p < 0.01. [file Image_3.tiff]

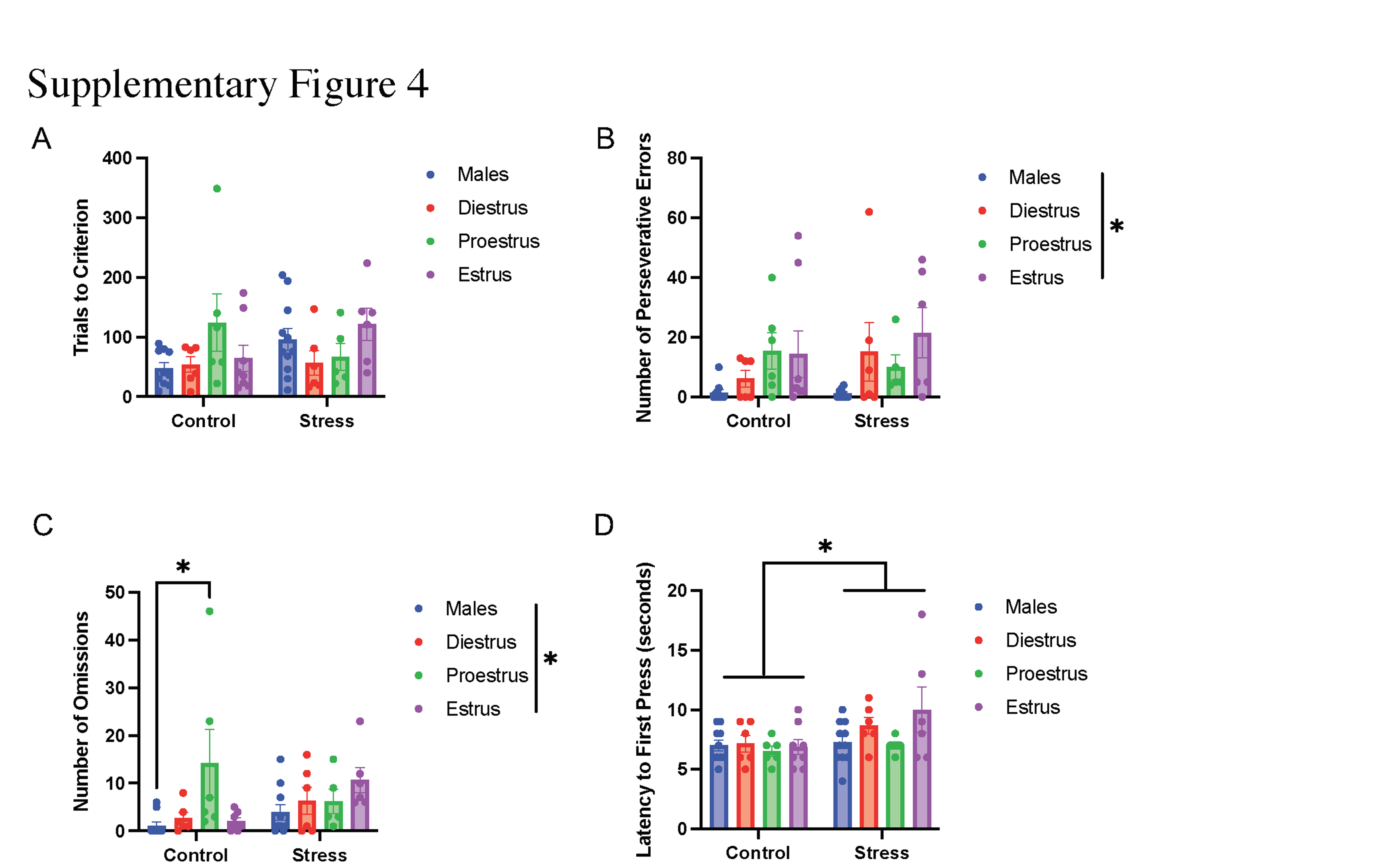

Supplement: Supplementary Figure 4 — Gonadal hormone status and acute stress impacted performance in the light discrimination task. (A) Gonadal hormone status and acute stress did not affect trials to criterion. (B) There was a main effect of gonadal hormone status on the number of perseverative errors; stressed female rats in the estrus phase made the highest number of errors. (C) There was a main effect of gonadal hormone status on the number of omissions; the control proestrus female rats had the highest number of omissions. (D) Stress increased the latency to first press. Two-way ANOVAs followed by Tukey post-hoc tests were conducted to examine the relationship between stress and gonadal hormone status, and the interaction between the two variables, on the measurements of light discrimination task performance for male (n = 22: control = 11, and stress = 11) and female (n = 40, control diestrus = 6; control proestrus = 6; control estrus = 8, stress diestrus = 8, stress proestrus = 6, and stress estrus = 6). Error bars are plotted as mean ± SEM. *p < 0.05. [file Image_4.tiff]

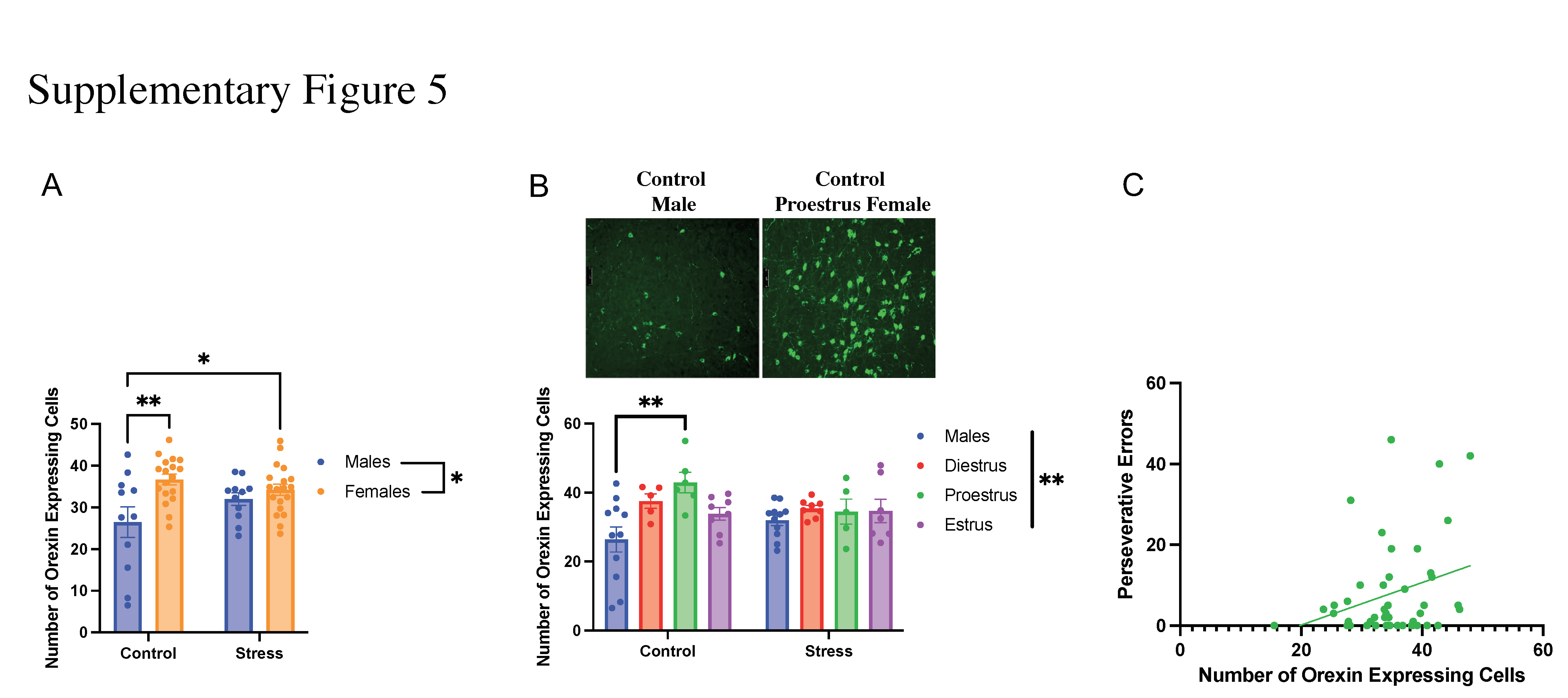

Supplement: Supplementary Figure 5 — Sex, gonadal hormone status, and stress affected the number of orexin-expressing cells. (A) There was a main effect of sex, and an interaction between sex and stress, on the number of orexin-expressing cells in the lateral hypothalamus. Specifically, the female rats in both the control and stress conditions had a higher number of orexin-expressing cells than the control male rats. (B) There was a main effect of gonadal hormone status, and an interaction between gonadal hormone status and stress, on the number of orexin-expressing cells; the proestrus female rats in the control group had more orexin-expressing cells than the male rats in the control group (representative image above of the number of orexin-expressing cells in the control male rats vs. the control proestrus female rats at Bregma level−3.3 mm). However, stress brought the male and female rats to comparable levels. (C) Number of orexin-expressing cells is positively correlated with perseverative errors in the light discrimination (LD) task. Two-way ANOVAs followed by Tukey post-hoc tests were conducted to examine the relationship between stress and sex or gonadal hormone status, and the interaction between the two variables, on the number of orexin-expressing cells for the males (n = 24, control = 12 and stress = 12) and female (n = 40: control = 20; stress = 20; control diestrus = 6; control proestrus = 6; control estrus = 8; stress diestrus = 8; stress proestrus = 6, and stress estrus = 6). A correlation was performed to determine the relationship between the number of orexin-expressing cells and perseverative errors in the light discrimination task. Error bars are plotted as mean ± SEM. *p < 0.05 and **p < 0.01. [file Image_5.tiff]

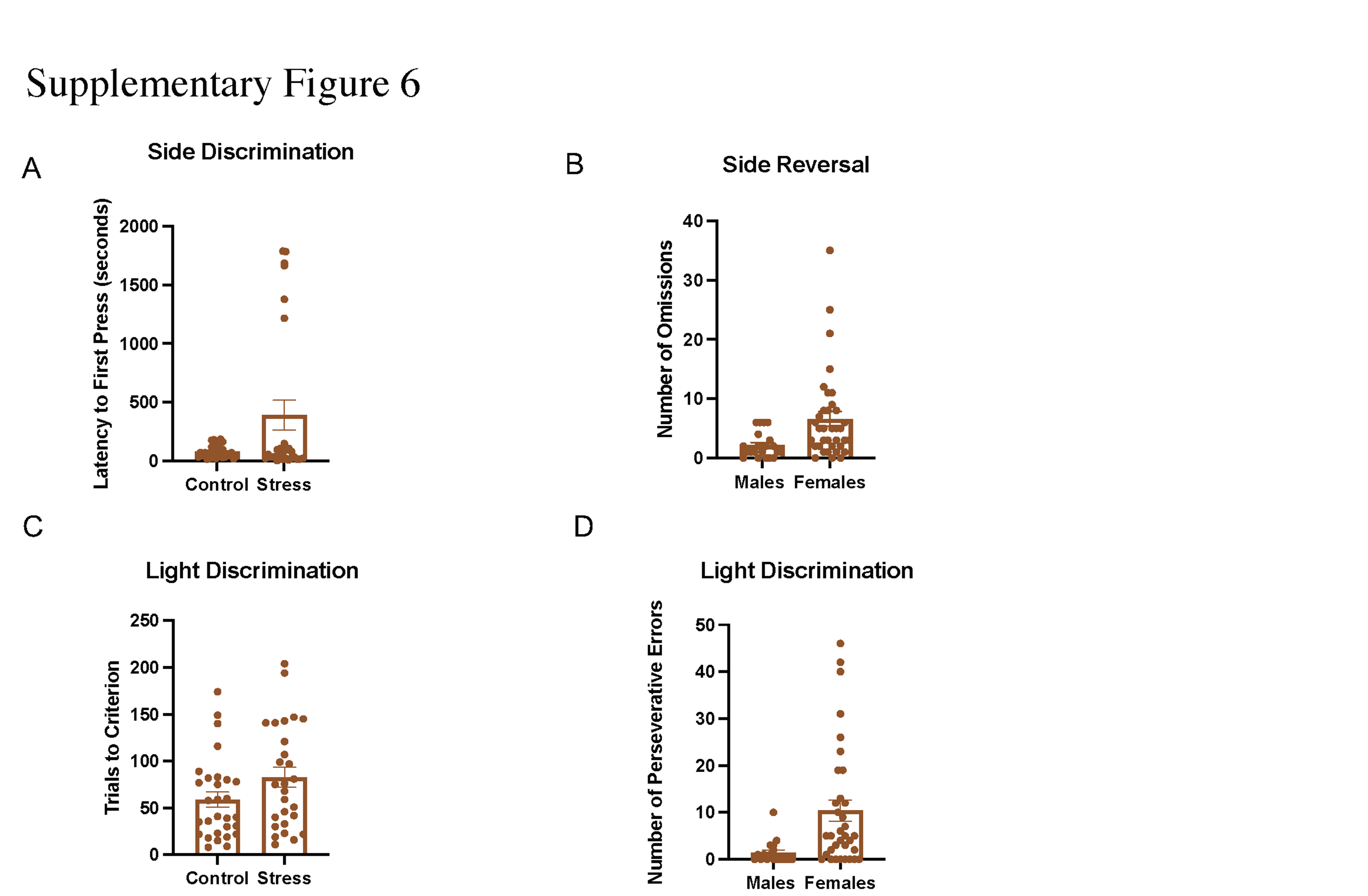

Supplement: Supplementary Figure 6 — Main effects of stress or sex displayed in graph format for original Figure 4 data. Acute stress and sex impacted performance in the side discrimination, side reversal, and light discrimination tasks. (A) Stress increased latency to first press in the side discrimination task. (B) Female rats exhibited a higher number of omissions than male rats in the side reversal task. (C) Acute stress increased trials to criterion in the light discrimination task. (D) Female rats demonstrated a higher number of perseverative errors in the light discrimination task than the male rats (n = 31 control, 30 stress; n = 21 male and 40 female rats). [file Image_6.tiff]

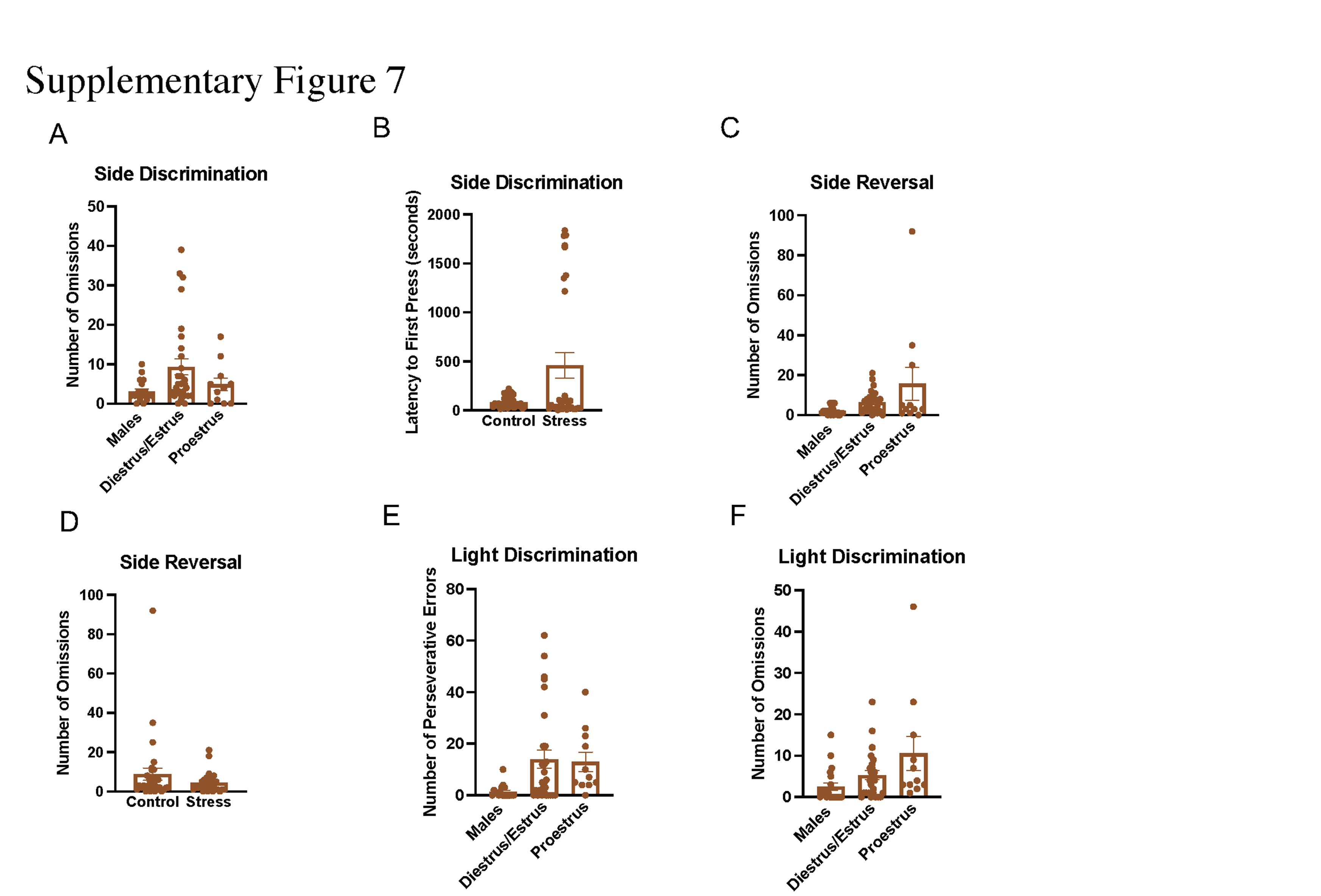

Supplement: Supplementary Figure 7 — Main effects of stress or gonadal hormone status displayed in graph format for original Figures 5–7 data. (A) Gonadal hormone status had a main effect on the number of omissions in the side discrimination task. (B) Stress increased the latency to first press in the side discrimination task. (C) Gonadal hormone status had a main effect on the number of omissions in the side reversal task. (D) Control animals demonstrated more omissions than stressed animals in the side reversal task. (E) Gonadal hormone status had a main effect on the number of perseverative errors in the light discrimination task. (F) Gonadal hormone status had a main effect on the number of omissions in the light discrimination task. (n = 31 control, 30 stress; n = 21 male, 28 diestrus/estrus female, and 12 proestrus female rats). [file Image_7.tiff]

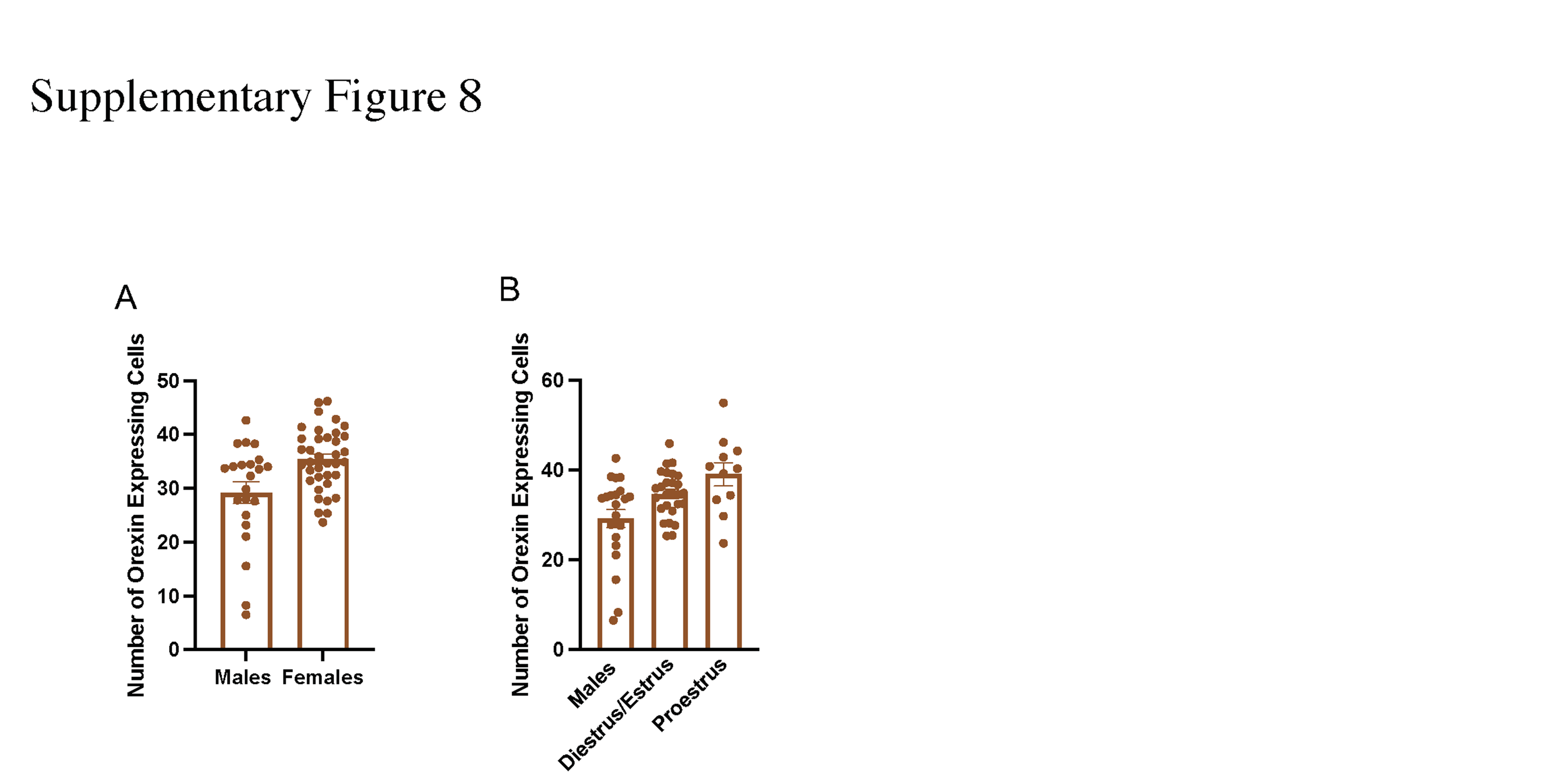

Supplement: Supplementary Figure 8 — Main effects of stress, sex, or gonadal hormone status displayed in graph format for original Figure 9 data. (A) Female rats have a higher number of orexin-expressing cells than male rats. (B) Gonadal hormone status has a main effect on the number of orexin-expressing cells in the lateral hypothalamus. (n = 24 male and 40 female rats; n = 24 male, 28 diestrus/estrus female, and 12 proestrus female rats). [file Image_8.tiff]
